# Association between sociodemographic factors and mobility among older adults: a systematic review and meta-analysis

**DOI:** 10.1186/s12877-026-06984-z

**Published:** 2026-01-16

**Authors:** Ogochukwu Kelechi Onyeso, David R. Scott, Chiedozie James Alumona, Michael E. Kalu, Israel Ikechukwu Adandom, Olayinka Akinrolie, Kelechi Mirabel Onyeso, Adesola C. Odole, Janice Victor, Jon Doan, Oluwagbohunmi A. Awosoga

**Affiliations:** 1 Faculty of Health Sciences, University of Lethbridge, Lethbridge, Alberta Canada; 2 Emerging Researchers and Professionals in Ageing-African Network, Abuja, Nigeria; 3 University of Lethbridge Library, Lethbridge, Alberta Canada; 4 School of Kinesiology and Health Science, Faculty of Health, York University, Toronto, Ontario Canada; 5 Department of Kinesiology, College of Education, The University of Alabama, Tuscaloosa, AL USA; 6 Lifespan Brain Health Laboratory, Department of Psychology, University of Calgary, Calgary, Alberta Canada; 7 Department of Estate Management, Faculty of Environmental Sciences, University of Nigeria, Nsukka, Enugu, Nigeria; 8 Department of Physiotherapy, Faculty of Clinical Sciences, College of Medicine, University of Ibadan, Ibadan, Oyo Nigeria

**Keywords:** Ageing, Community-dwelling, Gait speed, Mobility decline, Social determinants of health, Social gerontology, Socioeconomic

## Abstract

**Background:**

Mobility limitation is associated with poor quality of life, morbidity, and mortality among older adults. This pre-registered systematic review [PROSPERO CRD42022298570] synthesised the coefficients of association between sociodemographic factors and performance-based mobility outcomes in older adults (≥ 60 years).

**Methods:**

Electronic databases MEDLINE, WoS, EMBASE, CINAHL, AgeLine, and SPORTDiscus were searched from inception to 27 November 2023 for observational studies reporting an association between sociodemographic factors and performance-based mobility outcomes among older adults. Pairs of reviewers independently conducted title, abstract, and full-text screening, narrative synthesis, and meta-analysis following the PRISMA and MOOSE guidelines. The effect sizes, heterogeneity, dominance, and publication bias were analysed using R/R-Studio (version 4.3.2) and CMA (version 4).

**Results:**

Of the 9,328 studies screened, 57 were included (*n* = 130,060 participants); the pooled mean age was 69.81 ± 7.21years, habitual gait speed (HGS) = 1.01 ± 0.28 m/s, and time-up and go score = 7.67 ± 3.56s. The narrative synthesis showed that the majority of the studies found older age (92.2%), women (62.9%), non-Caucasian (75.0%), and lower education (64.5%) associated with significant mobility outcomes. There was a paucity of studies on marital status, area of residence, income, occupation, religion, homeownership, and social status. Meta-analysis showed that older age *r*=-0.37 [-0.42, -0.32] and female gender *r*=-0.13 [-0.22, -0.03] were moderately associated with slower HGS.

**Conclusion:**

Older age, female gender, non-Caucasian identity, and lower education were consistently associated with poorer mobility outcomes, pointing to sociodemographic sources of inequity and the need for targeted interventions. Limited evidence on marital status, residence, income, occupation, religion, homeownership, and social status restricted broader conclusions. Expanding research across these domains is critical to inform comprehensive strategies that advance equitable mobility and healthier ageing in diverse populations.

**Trial registration:**

Systematic review registration: PROSPERO CRD42022298570.

**Supplementary Information:**

The online version contains supplementary material available at 10.1186/s12877-026-06984-z.

## Background

Mobility is the ability of an individual to ambulate safely and independently, with or without a walking aid [1, 2]. Mobility is crucial for performing activities of daily living and impacts an individual’s independence and overall health [1, 3, 4]. Mobility limitation has dire consequences for older adults, including physical disability and institutionalisation [4], frequent falls and injuries [5], sedentary behaviour and dependency [3, 6], depression [7], social isolation [8], reduced quality of life [9], and death [4].

This review focuses on older adults aged 60 years and older [10]. The study is timely, as the global population of older adults is projected to reach 2.1 billion by 2050, placing unprecedented strain on the health, economic, and social systems of countries [10]. Several population-based studies have shown that mobility limitations in later life vary by sociodemographic characteristics such as age, sex or gender, income, education, and marital status [11–13]. Cohort evidence from studies such as the Health and Retirement Study in the United States of America [11], Longitudinal Ageing Study Amsterdam in the Netherlands [14], Ibadan Study on Aging in Nigeria [15], and the China Health and Retirement Longitudinal Study [16] suggests that lower socioeconomic position, limited educational attainment, non-Caucasian ethnicity, and being a woman are consistently associated with slower gait speed and an earlier onset of mobility limitations. However, findings across studies remain inconsistent because of differences in population composition, measurement tools, and analytic approaches, leaving uncertainty about the overall direction and magnitude of these associations [17].

There is a paucity of systematic literature reviews and meta-analyses on the broader sociodemographic determinants of mobility in older adults. Recent scoping reviews focused on cognitive, psychological, and environmental factors with little emphasis on social behaviour [18, 19]. Existing systematic reviews [20–22] mainly examined modifiable biophysical risk factors, treating sociodemographic variables as background descriptors rather than as core explanatory determinants. Therefore, a dedicated synthesis is needed to quantify these relationships and clarify social gradients in mobility, informing equity-focused ageing policy [17].

This review is guided by the life-course [23] and social determinants of health (SDH) [24] frameworks, which together explain how cumulative exposures and structural contexts shape health in later life. The SDH framework highlights how health inequalities, including mobility trajectories, are shaped by economic policies, development agendas, social norms, social policies, and political systems [24, 25], while the life-course perspective emphasises that early-life socially constructed experiences accumulate over time and influence functional capacity in old age [26]. Integrating these theories allows interpretation of mobility disparities as products of both lifelong accumulation and current social inequities.

Grounded on life-course and SDH theories, we conceptualised that mobility decline in older adults could be associated with cumulative sociodemographic influences, such as age, gender, marital status, area of residence, income, education, occupation, religion, social status, house ownership, and race or ethnicity [17]. Hence, we conducted a systematic review and meta-analysis of the association between sociodemographic factors and performance-based walking outcomes among community-dwelling older adults. The performance-based mobility outcomes included walk speed, time, and distance, measured through timed-up and go (TUG), habitual/usual gait speed (HGS), and time- and distance-specified walk tests [27]. Understanding the sociodemographic factors contributing to mobility decline among older adults is essential for formulating policies on ageing, allocating resources, and implementing geriatric interventions [28].

The research questions were as follows: (1) What is the direction of the association between sociodemographic factors and performance-based walking outcomes among community-dwelling older adults as reported in literature published from 1947 to 2023? and (2) What is the pooled effect size of the association between sociodemographic factors and performance-based walking outcomes among community-dwelling older adults?

## Methods

### Protocol and registration

The protocol [17] was registered within the International Prospective Register of Systematic Reviews (PROSPERO, CRD42022298570). The review was conducted and reported in adherence to the updated Preferred Reporting Items for Systematic Reviews and Meta-Analyses (PRISMA-2020) [29] and the Meta-analysis of Observational Studies in Epidemiology (MOOSE) guidelines [30]. The PRISMA-2020 checklist is provided in Supplementary File 1.

Due to the nature of the extracted data, we deviated from the published protocol as follows: (1) the pooled effect size was correlation coefficient (*r*) instead of odds ratio, (2) the risk of bias (ROB) assessment tool was changed from Prediction Model Risk of Bias Assessment Tool (PROBAST) to Joanna Briggs Institute’s (JBI) ROB appraisal checklist for analytic cross-sectional studies, (3) we conducted meta-analysis regardless of *I*^2^ statistics > 75%, (4) subgroup analysis of studies within 20-year intervals from 1947 to 2023 was not feasible because of limited number of included studies before 2009, (5) to facilitate the interpretation of the effect size (*r*), only zero-order and not partial correlation were included in the meta-analysis, and (6) the review timeline was amended and the search end date was extended from 28 February to 27 November 2023.

### Population, exposure, outcome, and timeline (PEOT) criteria

The study *population* was community-dwelling older adults aged 60 years and older. The *exposure* was sociodemographic factors: age, gender, marital status, area of residence, income, education, occupation, religion, social status, house ownership, and race or ethnicity. The *outcome* was performance-based walking outcomes: walking distance (m), time (s), and speed (m/s) obtained through timed-up and go test (TUG – 3 m and 8 ft), short physical performance battery (SPPB – gait speed component), timed walk test e.g. six-minute walk test (6MinWT), distance walk test e.g. ten-metre walk test (10MWT), and habitual gait speed (HGS) [27]. The review *timeline* was from the inception of the oldest included database (EMBASE-1947) to 27 November 2023.

### Eligibility criteria

#### Inclusion criteria

Studies were included if they (1) described an association between any sociodemographic variable and walk distance, walk time, or walk speed, (2) included older adult participants aged 60 years and older, or with a mean age of ≥ 60 years, and (3) were observational studies published in the English language between 1947 and 27 November 2023.

#### Exclusion criteria

Studies were excluded if (1) the target population were non-ambulatory, hospitalised, institutionalised, or continuing care facility residents, (2) conducted among older adults with specific disease conditions, including cancer, stroke, parkinsonism, Alzheimer’s disease, dementia, arthritis, sarcopenia, diabetes, chronic obstructive pulmonary disease, and organ failures, such as kidney or heart failure, and (3) the data were overlapping or a duplicate of an already included study.

### Information sources

Following Bramer and colleagues’ recommendation on electronic databases combination [31], MEDLINE (Ovid), EMBASE (Ovid), Web of Science, AgeLine (EBSCO), SPORTDiscus (EBSCO), and CINAHL (EBSCO) were searched from inception to 27 November 2023 by a health sciences librarian (DRS).

### Search strategy

**Fig. 1:**
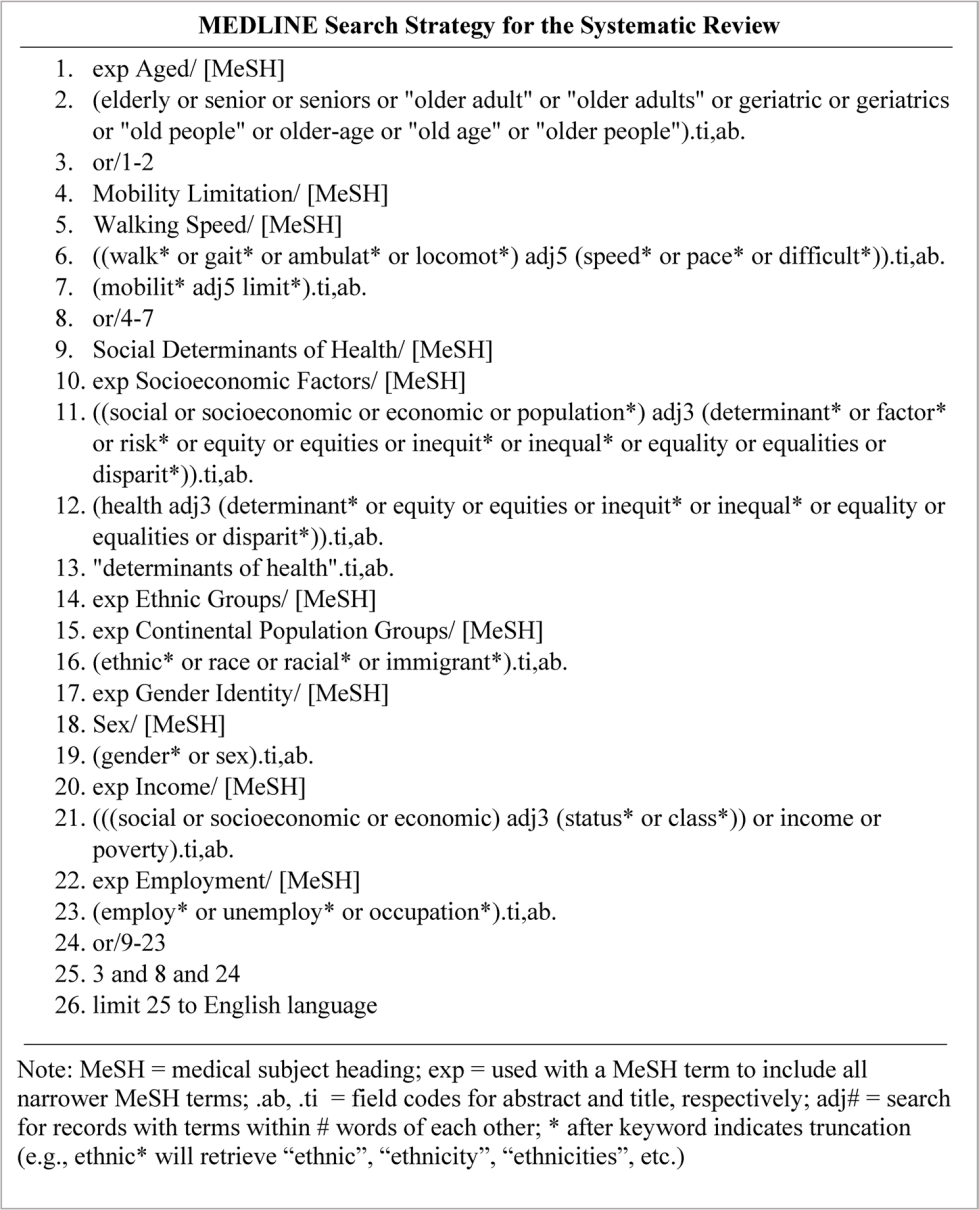
MEDLINE search strategy for the systematic review

Search terms were identified through consultations between the primary investigator (OKO), content experts (OAA, ACO, OA, MEK, JV, and JD), and the librarian (DRS), and a review of the titles and abstracts of six seed articles gathered by the primary investigator [12, 32–36]. Elements of search strings developed for previously published reviews also informed the search strategy [37–39]. Following the publication of the review protocol, the search strategy was reviewed by another librarian using the Peer Review of Electronic Search Strategies (PRESS) checklist [40], and several minor revisions were made based on the feedback provided. The search strategy was first developed for MEDLINE (Fig. 1) and then adapted for the other five databases (Supplementary File 2). When possible, subject headings from controlled vocabularies (e.g., MeSH) were used in the search. The search sensitivity was enhanced by entering concepts in the search string as keywords with truncation (e.g., ethnic*) and proximity operators (e.g., adj5) used when appropriate. Boolean operators connected subject headings and keywords, as shown in Fig. 1.

### Data management and study selection

The retrieved articles’ bibliographic information, including title, abstract, authors, publication metadata, and subject headings, was imported into EndNote 20 for deduplication [31]. After removing duplicate records, the articles were exported to Covidence, a systematic review management software [41], and screened in two phases: title/abstract and full-text screening. Two independent reviewers from the authorship list, randomly assigned by Covidence, completed the screening, with the software automatically assigning a third independent reviewer (OKO, CJA, IIA, or KMO) to resolve conflicts. Similarly, a pair of independent reviewers (OKO, CJA, IIA, or MEK) simultaneously completed the data extraction and risk of bias (ROB) assessment within Covidence using customised templates [17, 42]. The authors (OKO, CJA, IIA, and OA) hand-searched the reference lists of the included articles.

### Data items

A data extraction sheet, as outlined in the protocol by Onyeso et al. [17], was set up in Covidence and tested on a small sample of selected studies. The setup allowed two randomly paired reviewers (OKO, CJA, IIA, or MEK) to independently extract data, with OKO and CJA adjudicating any discrepancies. Consistent with the methodologies described by Lipsey and Wilson [43] and Khaliq et al. [44], we extracted key information from each study, including the first author’s surname, the year and country of publication, the study design (e.g., cohort, case-control, cross-sectional, or longitudinal), the sample size, the sociodemographic characteristics of participants, the mobility assessment tests used, and a descriptive summary of the outcomes. Additionally, we collected inferential statistical results, such as correlation and regression coefficients, effect sizes, standard errors, standard deviations, confidence intervals, and *p*-values.

### Risk of bias quality assessment

Two reviewers (OKO and CJA) independently evaluated the Risk of Bias (ROB) using the 8-item Joanna Briggs Institute’s (JBI) ROB appraisal checklist for analytic cross-sectional studies [42] and met to resolve any conflict. This checklist helped in assessing the internal validity of the studies, examining aspects such as the clarity of inclusion criteria, descriptions of participants and settings, measures of exposure, conditions and outcomes, confounding factors, and statistical analysis. Each item was rated as “Yes = 1”, “Unclear = 0”, or “No = 0”. The overall risk assessment was conducted by assigning each study a score from 0 to 8, with the total scores used to classify the studies into three ROB categories: High (0–3), Medium (4–5), and Low (6–8).

### Data analysis

The units of analysis were sociodemographic factors, mobility test, study design (cross-sectional and longitudinal studies), and type of inferential statistics (test of differences, bivariate, and multivariate association). A narrative synthesis was conducted to illustrate the direction of effects across all included studies. The synthesis for each sociodemographic factor was reported under the headings: bivariate analyses (including simple linear regression, Pearson’s correlation, and test of differences, such as t-test and analysis of variance [ANOVA]) and multivariate analyses.

The meta-analysis included only studies that reported a bivariate association (zero-order correlation) between a sociodemographic factor and walking parameter or provided sufficient information to calculate these associations [31]. The Comprehensive Meta-Analysis (version 4) [45] and R software (version 4.3.2, ‘metafor’ and ‘robumeta’ packages) [46] were used for quantitative synthesis. The overall estimate was computed for studies with overlapping samples. In cases where studies reported effect sizes from independent subgroups, each subgroup was treated as a separate sample in the meta-analysis [47]. The effect sizes (correlation coefficients) were transformed to Fisher’s z-scores to adjust for normal distribution [46]. The inverse variance method was employed, which reduces the variance of the weighted average of effect sizes [47, 48]. The pooled mean effect size was then calculated using a random-effects model [47], which is suitable for drawing statistical inferences from studies of a heterogeneous population and generalising beyond the included studies [49]. The pooled estimates were presented using Forest plots.

Heterogeneity was evaluated by visually examining the Forest plots, the *p*-value of Cochran’s Q test (*p* < 0.05), and the *I*^2^-statistic value. An *I*^2^ statistic of 0% indicates no heterogeneity, 50% indicates moderate heterogeneity, and 75% indicates high heterogeneity [50]. The influence analysis, Cook’s distance (*D*), was calculated to identify studies with overt dominance in the meta-estimates; a threshold of *D* > 0.5 was used to qualify a study as influential [46]. Where necessary, “one-study-removed” sensitivity analysis was applied to reduce subgroup heterogeneity, considering effect sizes outside the 95% confidence interval of the mean effect size as outliers [45]. Publication bias was assessed using three indicators: funnel plot, Rosenthal’s Fail-Safe N, and Egger’s regression intercept.

## Results

### Study selection

**Fig. 2:**
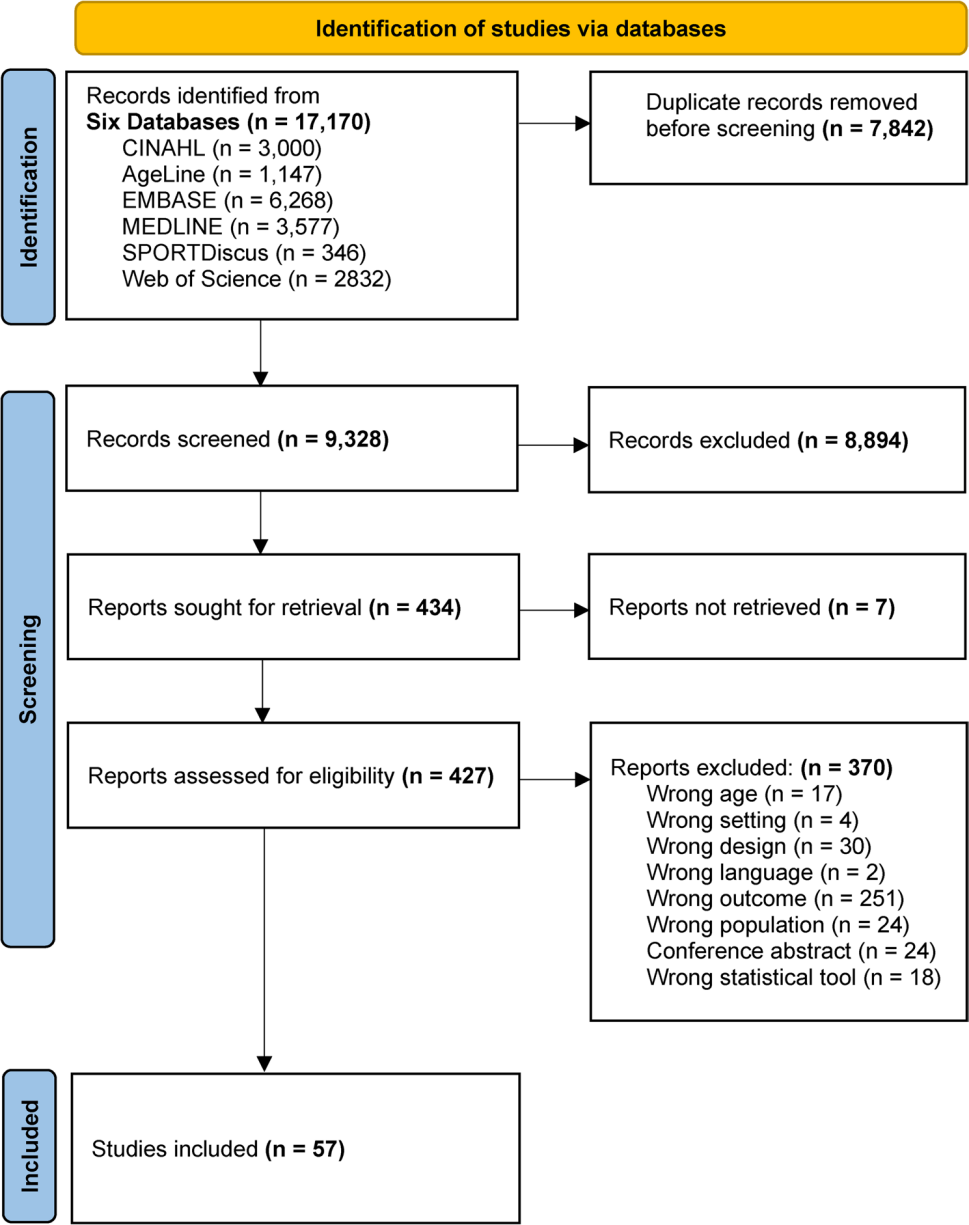
PRISMA flowchart

The search across six databases yielded 17,170 results, of which 7,842 duplicates were removed. The PRISMA flowchart (Fig. 2) details the screening process of 9,328 abstracts, leading to the exclusion of 8,910 studies that did not meet the inclusion criteria. Seven of the remaining 428 articles could not be retrieved through the library’s collection or interlibrary loan. The 421 retrieved full texts were assessed, and 57 were selected for analysis. No additional articles were found through hand-searching their references.

### Study characteristics

**Fig. 3:**
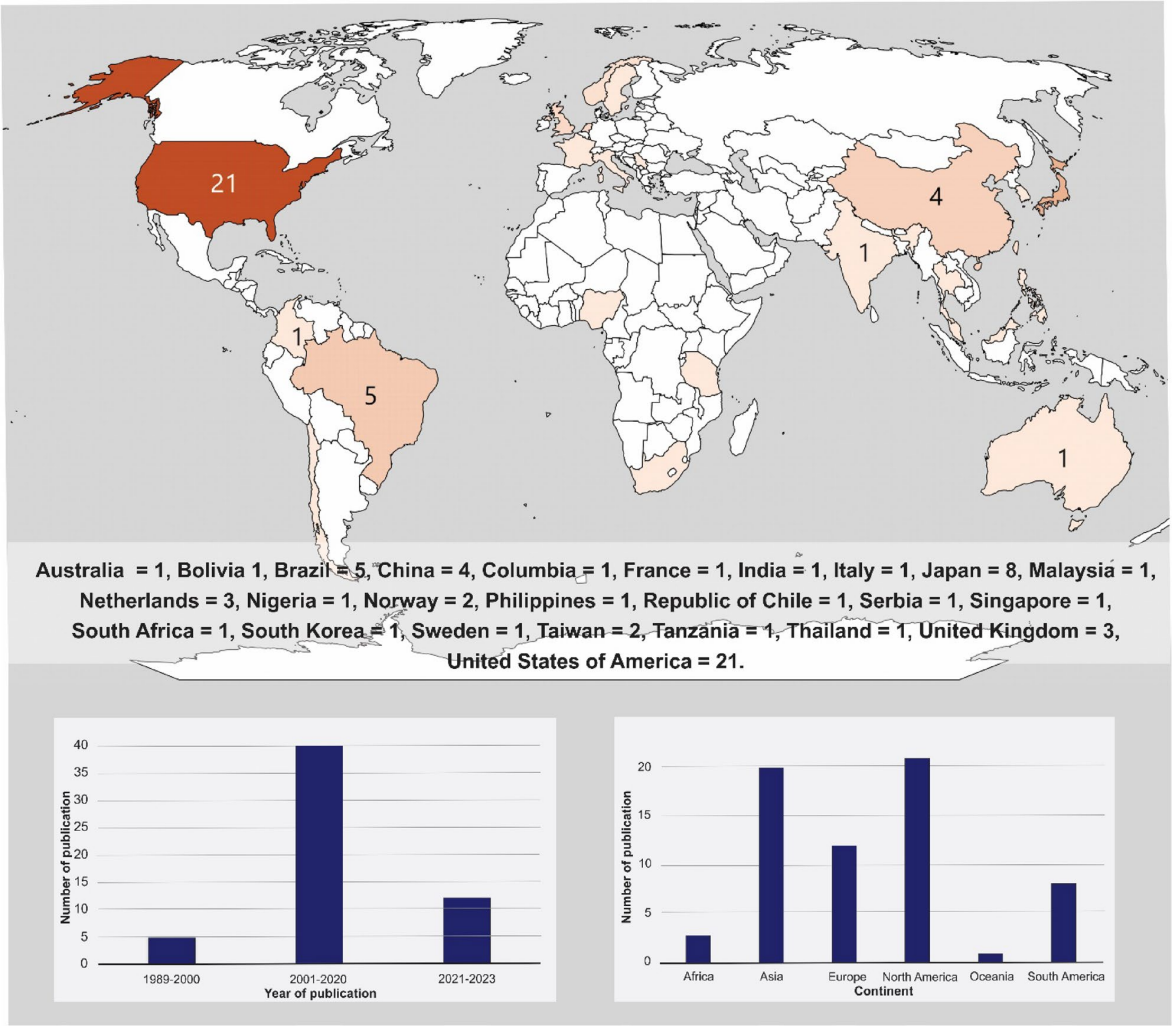
Periods and countries where included studies were conducted

The studies were conducted across 25 countries between 1989 to 2023 (Fig. 3). The studies’ characteristics (Supplementary File 3) showed that 50 employed cross-sectional analyses, five were longitudinal, and two employed both designs. The review involved a total sample of 130,060 participants with a pooled mean ± SD age of 69.81 ± 7.21 years (95% CI: 69.77–69.85), ranging from 60.25 to 80.90 years. The sample was 58.2% women, 60.1% married, and 72.7% identified as Caucasian. The pooled average HGS = 1.01 ± 0.28 m/s (95% CI: 1.01, 1.05), range 0.52 to 1.37 and TUG = 7.67 ± 3.56 s (95% CI: 7.61, 7.73), range 5.71 to 12.6, reflected the general mobility status of the study populations. Table 1 summarised the narrative synthesis, indicating that 92.1% of the unique analyses found that older age was associated with mobility limitations. Other significant factors included being female (64.7%), non-Caucasian (62.5%), and having lower educational attainment (64.5%). Studies on marital status, area of residence, income, occupation, religion, homeownership, and social status were few and presented inconsistent results (Supplementary File 4). Only some studies addressing age and gender met the criteria for meta-analysis, as highlighted in the data analysis section.

**Table 1:** Summary of the narrative synthesis of the association between sociodemographic factors and mobility outcome

Result	Cross-sectional analysis f(%)		Longitudinal analysis f(%)		Total f(%)
	Bivariate	Multivariate	Bivariate	Multivariate	
Age					
Older age → significantly lower mobility	39 (92.9)	26 (92.9)	2 (66.7)	4 (100.0)	71 (92.2)
Younger age → significantly lower mobility	-	-	-	-	-
The result was not statistically significant	3 (7.3)	2 (7.1)	1 (33.3)	-	6 (7.8)
Gender					
Men → significantly higher mobility	24 (63.2)	18 (66.7)	1 (33.4)	1 (50.0)	44 (62.9)
Women → significantly higher mobility	1 (2.6)	-	1 (33.3)	-	2 (2.9)
The result was not statistically significant	13 (34.2)	9 (33.3)	1 (33.3)	1 (50.0)	24 (34.2)
Marital status					
Married/has partner → significantly higher mobility	1 (100.0)	1 (50.0)	1 (100.0)	-	3 (75.0)
Has no partner → significantly higher mobility	-	-	-	-	0 (0.0)
The result was not statistically significant	-	1 (50.0)	-	-	1 (25.0)
Race					
Caucasians → significantly higher mobility	7 (77.8)	4 (100)	1 (33.3)	-	12 (75.0)
Non-Caucasians → significantly higher mobility	1 (11.1)	-	-	-	1 (6.2)
The result was not statistically significant	1 (11.1)	-	2 (66.7)	-	3 (18.8)
Education level					
Higher education → significantly higher mobility	14 (93.3)	6 (60.0)	-	-	20 (64.5)
Lower education → significantly higher mobility	-	1 (10.0)	1 (33.3)	1 (33.3)	3 (9.7)
The result was not statistically significant	1 (6.7)	3 (30.0)	2 (66.7)	2 (66.7)	8 (25.8)
Income level					
High income → significantly higher mobility	1 (50.0)	2 (100.0)	-	1 (100.0)	4 (80.0)
Low income → significantly higher mobility	-	-	-	-	-
The result was not statistically significant	1 (50.0)	-	-	-	1 (20.0)
Occupation type					
Manual job → significantly higher mobility	-	-	-	-	-
Non-manual job → significantly higher mobility	2 (66.7)	-	-	-	2 (66.7)
The result was not statistically significant	1 (33.3 )	-	-	-	1 (33.3 )
Area of residence					
Urban residents → significantly higher mobility	1 (100.0)	-	-	-	1 (50.0)
Rural residents → significantly higher mobility	-	1 (100.0)	-	-	1 (50.0)
The result was not statistically significant	-	-	-	-	-
Homeownership					
Homeowners → significantly higher mobility	-	-	-	1 (100.0)	1 (100.0)
Non-homeowners → significantly higher mobility	-	-	-	-	-
The result was not statistically significant	-	-	-	-	-
Social status					
High social status → significantly higher mobility	-	1 (100.0)	1 (100.0)	-	2 (66.7)
Low social status → significantly higher mobility	-	-	-	-	-
The result was not statistically significant	1 (100.0)	-	-	-	1 (33.3)
Religious participation	-	-	-	-	

*NB* The total number of studies included was 57, but some analysed multiple independent datasets

### Study risk of bias

**Fig. 4:**
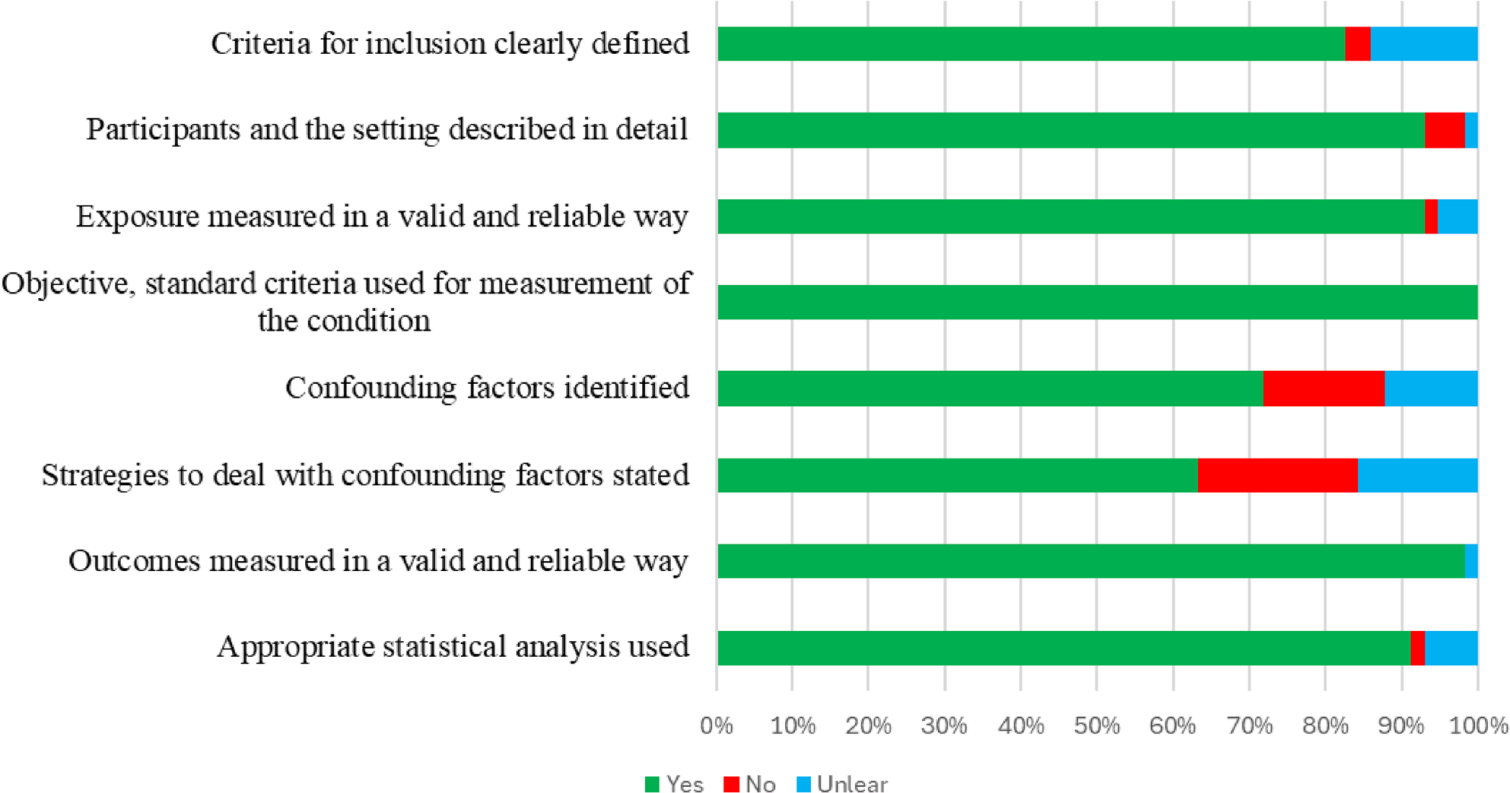
Joanna Briggs Institute’s checklist summary of the risk of bias of all included studies

The overall ROB of the included studies is presented in Fig. 4. Detailed ROB evaluations for each study are presented in Supplementary File 5. Among the 57 included studies, 51 (89.5%) exhibited a low ROB, while six (10.5%) demonstrated a moderate ROB, with none categorised under high risk of bias. The average ROB score was 7.

### Associated sociodemographic factors

#### Age

##### Bivariate analysis

Forty-nine of the 57 studies reported the influence of age on older adults’ mobility. Older age was significantly associated with slower HGS, as reported in 96.4% of the cross-sectional bivariate analyses [11, 13, 15, 32–35, 51–70]. Similarly, poorer TUG performance [51, 59, 68, 69, 71–75] and 6MinWT performance [59, 65, 72] were significantly associated with older age. However, three studies found no significant age-group difference in HGS [76] and TUG scores [69, 77]. Two longitudinal bivariate analyses reported a significant decline in TUG among the older age groups after six [78] and nine-year follow-ups [79], and no significant difference in HGS was found [78].

##### Multivariate analysis

Cross-sectional multivariate analyses showed that older age was significantly associated with slower HGS [11, 13, 32, 35, 36, 54, 56, 57, 59, 61, 63, 66, 68, 80–86], poorer TUG [59, 68, 73, 87, 88], and 6minWT performances [65]. Two cross-sectional studies found no significant multivariate association between HGS and age [67, 89]. However, longitudinal multivariate analyses showed a significant association between age and mobility (HGS and TUG) decline after a three-year [90, 91], five-year [92], and nine-year [79]follow-up.

##### Meta-analysis

**Fig. 5:**
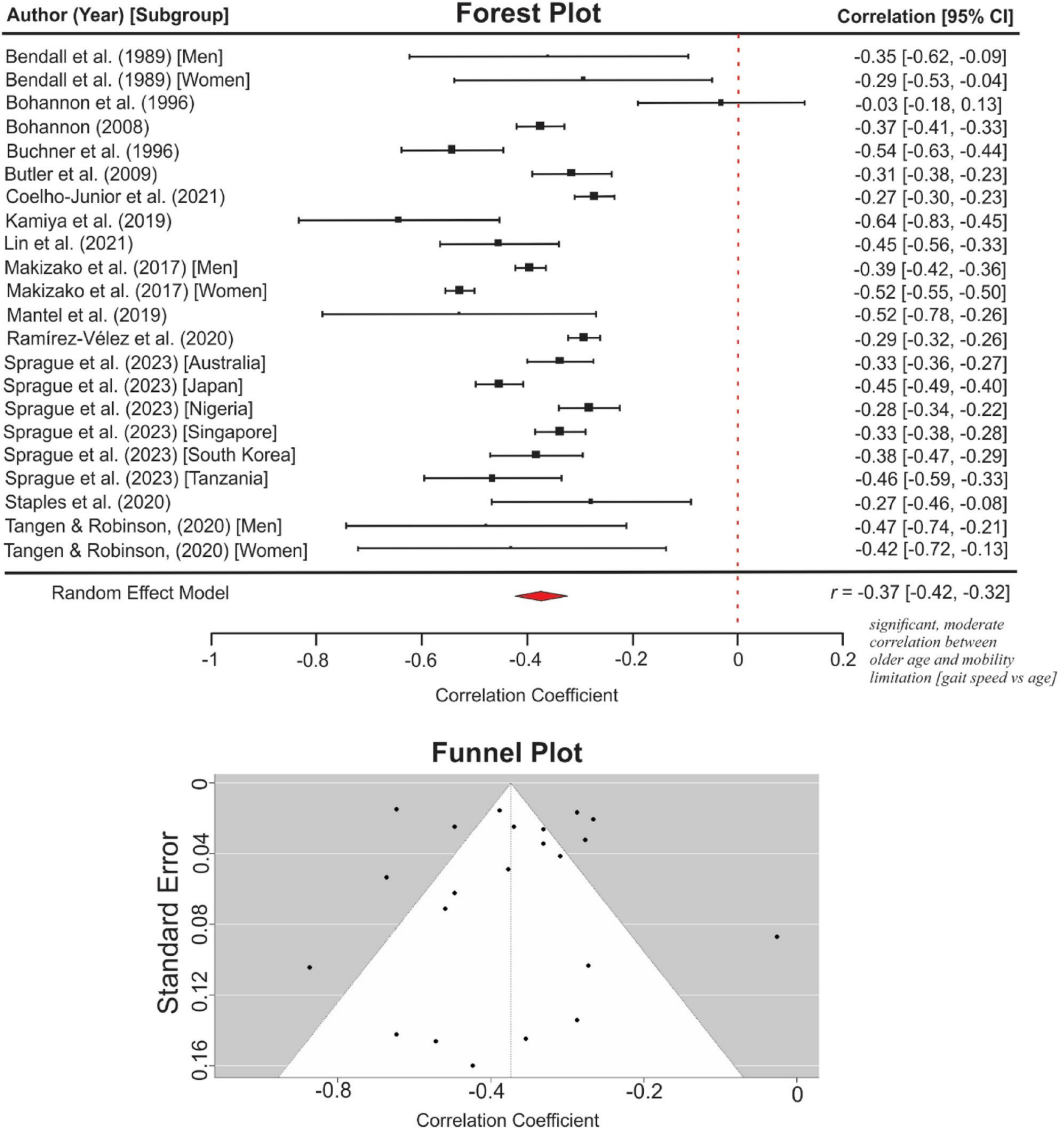
Meta-analysis of the association between age and habitual gait speed

The meta-analysis of 22 cross-sectional bivariate analyses (Fig. 5) showed that older age correlated significantly with slower gait speed. The pooled effect size (*r*) = -0.37 (95% CI: -0.42, -0.32), *p* < 0.001. Cochran’s *Q* = 253.68, *p* < 0.001, *I*^2^ = 92.1% suggests substantial heterogeneity among the studies. Rosenthal’s Fail-Safe (*N*) = 35.14 and Egger’s Regression Intercept (*b*) = -0.388, *p* = 0.763, show no evidence of publication bias. None of the studies overtly influenced the pooled estimate (*D* < 0.5).

The “one-study-removed” sensitivity analyses produced no significant difference in the heterogeneity indices. However, the simultaneous removal of seven studies [15, 51, 56, 62, 64, 68, 76] whose estimate fell outside the 95% CI, yielded a remarkable difference in heterogeneity, *Q* = 159.54, *p* < 0.001, *I*^2^ = 71.7% and effect estimate *r* = -0.42 (95% CI: -0.49, -0.38), *p* < 0.001. There was no statistical evidence of publication bias; Rosenthal’s *N* = 80.55 and Egger’s *b* = -0.448, *p* = 0.554.

**Fig. 6:**
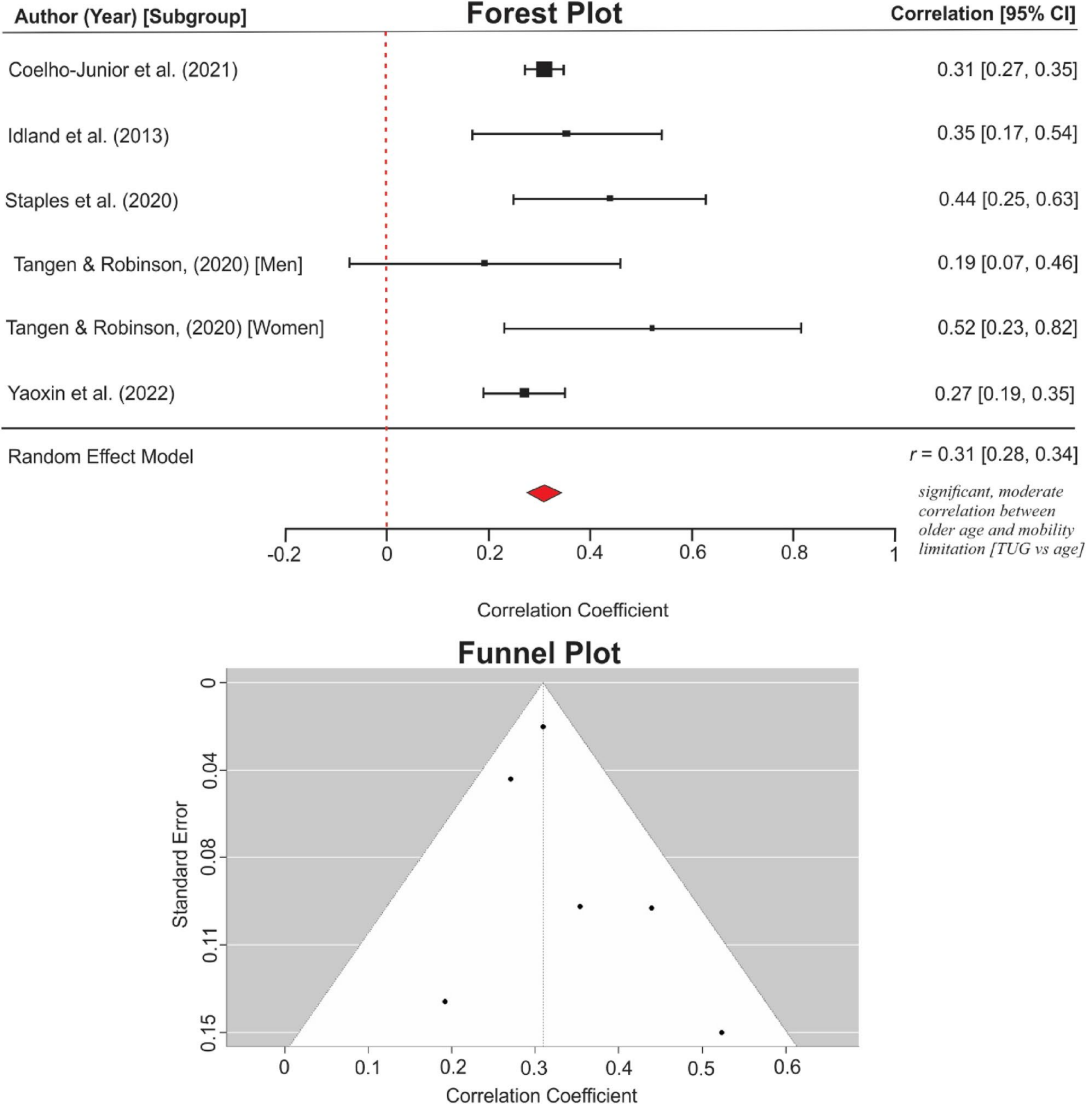
Meta-analysis of the association between age and timed-up and go score

The meta-analysis of six studies reporting the effect of age on TUG (Fig. 6) showed a pooled effect size *r* = 0.31 (95% CI: 0.28, 0.34), *p* < 0.001, with a low heterogeneity *Q* = 5.73, *p* = 0.333, *I*^2^ = 0.1% and no statistical evidence of publication bias (Rosenthal’s *N* = 85.64 and Egger’s *b* = 0.292, *p* = 0.459). One study [75] with a high correlation coefficient (*r* = 0.81, *n* = 100) was excluded from the analysis as a severe outlier. The longitudinal analysis subgroups for HGS and TUG did not meet the criteria for meta-analysis.

#### Gender or sex at birth

##### Bivariate analysis

Forty-five of the 57 studies reported the effect of gender or sex at birth on older adults’ mobility. Most cross-sectional bivariate analyses reported significantly faster HGS for men compared with women [11, 13, 15, 33, 34, 54, 56, 57, 59–64, 70, 76, 86, 89]. However, Sprague et al. [15] found the reverse in a Japanese dataset. The rest of the studies found no significant difference [15, 32, 35, 52, 53, 58, 66, 67, 93, 94]. For walk distance, three studies reported that men covered longer distances during 6MinWT [59, 65, 72], but Kamiya et al. [65] result was not significant. Cross-sectional bivariate analyses showed that men had better TUG scores than women [59, 72, 73, 77], but two studies found the outcome not statistically significant [71, 75]. Two longitudinal bivariate analyses of HGS decline gave contrasting results. Gomes et al. [78] six-year follow-up found women, but Sialino et al. [14] 15-year follow-up found men to have more HGS decline. However, Gomes et al. [78] found no statistically significant gender differences in TUG decline.

##### Multivariate analysis

Most of the multivariate analyses showed that being a man was significantly associated with faster HGS [11, 13, 16, 36, 54, 56, 57, 59, 61, 63, 76, 80, 82, 94]. The remaining studies found no statistically significant gender effect [66, 67, 69, 83, 84, 89]. In terms of the time of completion of walking tasks, three cross-sectional analyses showed that being a man was significantly associated with a shorter time [59, 73, 87], while two studies found no significant gender association [69, 88]. Similarly, Thaweewannakij et al. [59] cross-sectional analyses found male gender as a significant predictor of a longer walking distance among older adults, while Kamiya et al. [65] found the same effect direction, but not statistically significant. In a 15-year longitudinal multivariate model, Sialino et al. [14] found a significant association between HGS decline and woman gender. Vasunilashorn et al. [91] reported no statistical significance after a three-year follow-up.

##### Meta-analysis

**Fig. 7:**
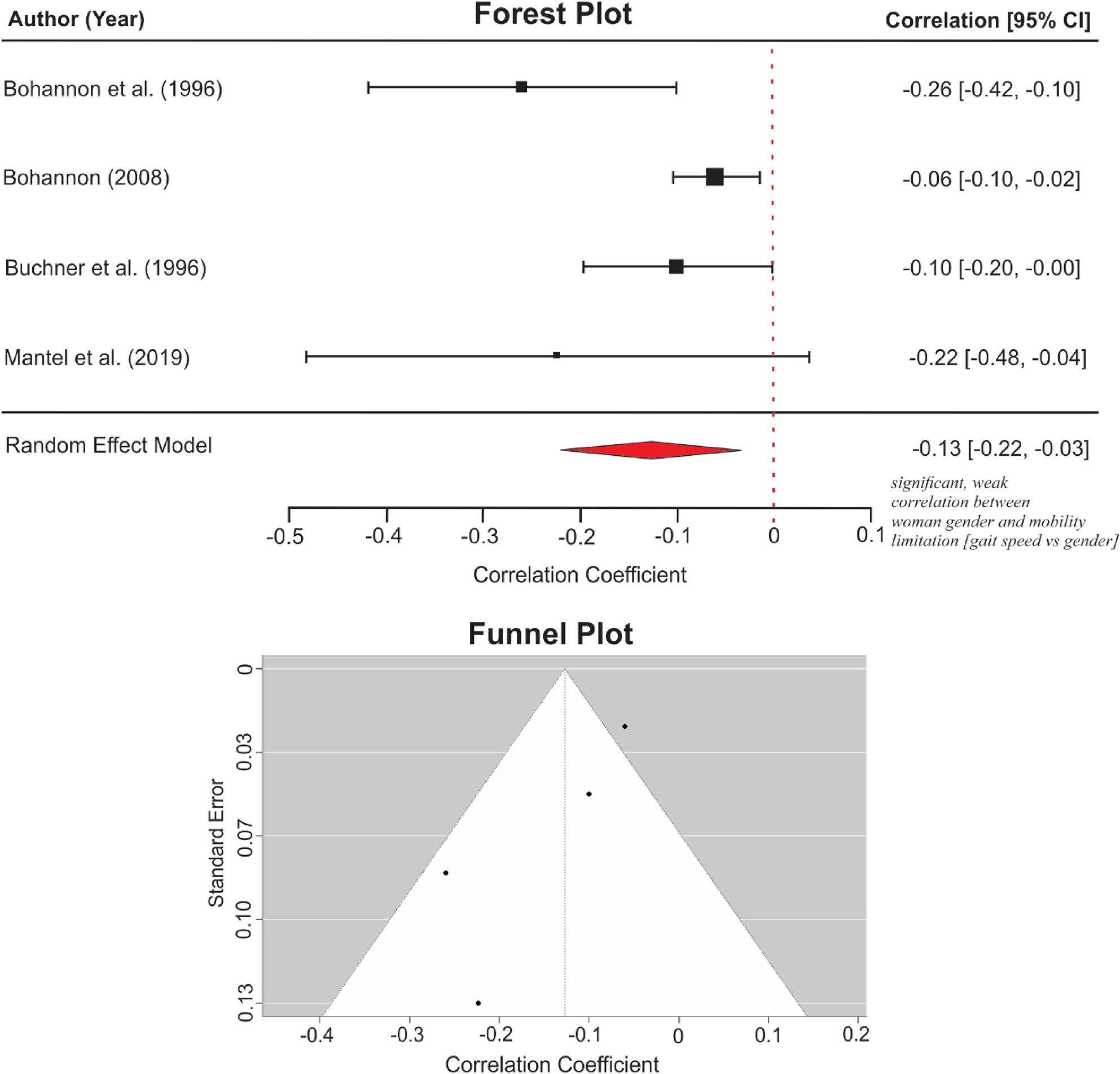
Meta-analysis of the association between gender and habitual gait speed

The meta-analysis of four cross-sectional bivariate studies suggested that being a woman was associated with slower HGS. The pooled effect size *r* = -0.13 (95% CI: -0.22, -0.03), *p* = 0.007 (Fig. 7). There was a moderate heterogeneity *Q* = 7.03, *p* = 0.071, *I*^2^ = 62.6%, and no statistical evidence of publication bias; Rosenthal’s *N* = 2.12 and Egger’s *b* = -0.011, *p* = 0.095. Only the cross-sectional bivariate HGS subgroup met the criteria for inclusion in the meta-analysis.

#### Marital status

##### Bivariate analysis

Four of the 57 included studies reported the influence of marital status on mobility among older adults. A cross-sectional [34] and longitudinal [78] bivariate analysis reported that those who were married or with partners had significantly better HGS than those without partners or unmarried. Furthermore, Gomes et al. [78] reported that people with no partner had a more significant decline in TUG scores.

##### Multivariate analysis

Cross-sectional multivariate analyses conducted by Payne et al. [13] and Al Snih et al. [87] showed a positive influence of having a partner or being married on the HGS and 8ft walk test, respectively. Payne et al.‘s [13] result was statistically significant, but Al Snih et al. [87] was not.

#### Race

##### Bivariate analysis

Twelve studies conducted a race-based analysis of mobility in older adults. Five bivariate cross-sectional analyses showed that Caucasians had significantly better HGS [11, 34, 36, 60, 84, 93], but a study reported no significant results [35]. Conversely, a study reported that the Japanese had better HGS than Caucasians [95]. Regarding timed walk tests, cross-sectional bivariate analysis [87] reported that Caucasians had significantly better 8ft WT scores than non-Caucasians. A six-year longitudinal analysis by Gomes et al [78] showed that Caucasians had a lesser but not statistically significant HGS and TUG decline. However, Thorpe et al [92] showed that Caucasians had significantly less HGS decline in a five-year longitudinal analysis. 

##### Multivariate analysis

All the cross-sectional multivariate analyses – four studies reported that being a Caucasian was significantly associated with better HGS relative to other races, especially Blacks [11, 81, 84, 93]. No study reported a race-based longitudinal multivariate analysis.

#### Education

##### Bivariate analysis

Twenty-two of the 57 studies reported the impact of education on mobility in older adults. About 93% of the cross-sectional bivariate analyses showed that higher education attainment was significantly associated with better HGS [32, 33, 35, 52, 54, 60, 61] and TUG scores [71, 96]. Additionally, Sprague et al. [15] analysed six datasets and found higher education associated with better HGS, except for a Nigerian dataset that showed no significant result. A six-year follow-up longitudinal bivariate analysis reported that people with higher education had a significantly higher HGS decline, but less and non-significant TUG decline [78]. Similarly, Idland et al. [79] reported that people with lower education had a lower TUG decline, but this was not statistically significant.

##### Multivariate analysis

Six cross-sectional multivariate analyses showed that higher education was significantly associated with better HGS [11, 33, 35, 36, 54] and TUG scores [87], but three studies found no significant association between educational attainment and HGS [13, 81, 84]. Conversely, Staples et al. [68] reported that higher education was associated with poorer TUG performance. A five-year longitudinal multivariate analysis showed that higher education was significantly associated with less HGS decline [92], while Vasunilashorn et al.‘s [91] three-year and Sialino et al.‘s [14] 15-year follow-up found no significant association.

#### Income

##### Bivariate analysis

Five studies reported the influence of income on the mobility of older adults. One cross-sectional bivariate analysis reported that high-income earners had significantly better HGS than low-income earners [34], while another study found no significant association between income and HGS [32].

##### Multivariate analysis

Two cross-sectional multivariate analyses showed that high-income earners had significantly better HGS than low-income earners [11, 81]. A five-year longitudinal multivariate analysis showed that low income significantly predicted HGS decline [92].

#### Occupation

Three cross-sectional studies reported the association of manual (unskilled) and non-manual (skilled) jobs with mobility in older adults. Welmer et al. [33] (bivariate) and Plouvier et al. [12] (multivariate) reported significantly higher HGS in older adults with non-manual than manual occupations. However, bivariate analysis by Yaoxin et al. [71] found no significant job-type-related differences in TUG scores.

#### Area of residence

Two cross-sectional studies reported contrasting findings on the implications of urban and rural residence on older adults’ mobility. Compared to rural residents, urban residents had significantly higher HGS and 6MinWT scores in bivariate analyses [55] or slower gait speed within a multivariate-adjusted model [11].

### Homeownership

One study reported a five-year follow-up longitudinal multivariate analysis showing that not owning one’s home was significantly associated with HGS decline [92].

### Social status

Only three of the included studies reported the impact of social status on mobility. A cross-sectional bivariate analysis showed that people with higher social status had better HGS, though the outcome was not statistically significant [34]. However, a six-year follow-up longitudinal bivariate analysis reported a significant decline in HGS and TUG in older adults with low relative to high socioeconomic status [78]. Similarly, a 15-year follow-up longitudinal multivariate analysis showed that lower social participation was associated with significant HGS decline [14].

## Discussion

The review examined the direction and magnitude of the association between sociodemographic factors and performance-based mobility outcomes among older adults. Concerning the direction of the association, the findings indicate that older age, female gender, single or unpartnered status, non-Caucasian race, lower education, and lower income were significantly associated with poorer mobility performance. Three or fewer studies addressed the impacts of occupation type, area of residence, homeownership, and social status; however, the findings across these studies were inconsistent, thus limiting our ability to draw reliable conclusions. No studies on religious participation met the inclusion criteria.

Regarding effect magnitude, meta-analyses of cross-sectional bivariate results showed that older age and being a woman were moderately correlated with lower mobility scores. However, meta-analysis for other sociodemographic factors could not be conducted, as the included studies did not meet the required criteria: a sufficient number of studies reporting zero-order associations, using the same research design and statistical method, and comparable performance-based measures. Heterogeneity (*I*^*2*^-statistics) was negligible in the TUG-based meta-analysis for the effect of age (0.1%), but moderate in the HGS-based analyses for age (71.7% after outlier removal) and gender (62.6%). The TUG is generally considered a more stable measure of functional mobility in older adults than HGS [27, 97, 98]. Moreover, the higher heterogeneity observed in the HGS analyses may be attributed to variations in test calibration and protocols. For instance, although gait speed was consistently reported in metres per second, studies used walk paths of varying lengths, including 2.4 m [66], 3.7 m [67], or 4 m paths [69]. No evidence of publication bias was detected across analyses based on Egger’s test, Rosenthal’s Fail-Safe test, and visual inspection of funnel plots. Nevertheless, tests for publication bias could be imprecise when there are fewer than ten studies [99]. Therefore, the TUG-age and HGS-gender analyses should be interpreted with caution.

From the life-course and SDH perspective, these results illustrate how cumulative social exposures and structural contexts shape functional capacity in later life [23, 26, 37]. The findings were interpreted through the lens of the socio-determinants of health, providing an alternative perspective to the biophysical models [100]. It is essential to note that three of the eleven sociodemographic factors in this review: age, sex, and race, can be explained through socially constructed and biophysical mechanisms. The physiological implications of old age, sex, and race have been well-established within the biophysical models; however, social scientists argue for a social perspective that addresses how the cumulative effects of ageism, sexism, and racism can be superimposed on the biophysical model.

Human ageing naturally leads to biological decline, including mobility limitation. The strong association between age and mobility decline reflects not only biological ageing but also the lifelong accumulation of social and environmental constraints that limit opportunities for physical activity and health maintenance. Ageist actions, including the medicalisation of old age, older adult abuse and neglect, forced retirement, institutionalisation, and systematic exclusion during policymaking, can negatively impact the mobility potentials of older adults [101, 102]. Governments should develop national policies for healthy ageing, including social security, rights protection and caregiving support for older adults [103]. For example, incorporate standardised mobility assessments such as TUG, HGS, and balance tests into annual check-ups for older adults, enabling early detection of functional decline and fall risk [104]. Similarly, geriatric practice guidelines should integrate social prescribing modes that prompt clinicians to refer older adults to community-based physical activity programmes, walking groups, or intergenerational engagement initiatives that promote movement and reduce isolation.

Sex- and gender-related findings align with the life-course concept of cumulative role strain. Sex differences in anatomy and physiology are evident, with biophysical impacts often emerging at older age [16, 105, 106]. Women often experience a double burden of domestic labour and occupational demands, resulting in sustained physical and psychosocial stress that may manifest as mobility limitations in later life. Some socioculturally constructed gender roles, including unpaid and unrecognised labour relating to childbirth, childrearing, housekeeping, and discriminatory practices, may predispose women to earlier mobility decline than men [14, 107]. To mitigate the life course impact of some socially constructed women’s work overload, we concur with Yavorsky et al. (2015) on the spousal division of labour. Formal interventions should target gender and work structures by supporting women through community groups, flexible workplace policies to lower maternal strain, and fair retirement benefits.

Race is another social construct with biological intricacies. Some racial differences may have genetic basis in human senescence, but racism creates systematic disadvantages that can affect older adults’ mobility, as seen between American Blacks and Caucasians [92]. Racial and ethnic disparities in mobility can also be understood within the SDH framework as products of structural inequities. Differential access to education, income, and healthcare, together with systemic racism, can cumulatively disadvantage racialised groups, limiting their capacity to maintain physical function. In a multiracial society, Caucasians, especially males, often occupy positions of privilege, affording them better access to health, financial resources, social status, and a higher quality of life [108, 109]. Conversely, an older Black woman faces compounded discrimination due to ageism, sexism, and racism, creating additional structural disadvantages [92]. Beyond racism, racial identity aligns with cultural practices, such as dietary patterns [110], health-seeking behaviours [111] and beliefs on healthy ageing [112], which may influence older adults’ mobility. However, investment in education can mitigate the impacts of racism and cultural disparities through its effects on socioeconomic status and health literacy [111].

Socioeconomic factors, including education [33, 113], occupation [12, 114], and income [115], affect mobility independently and collectively [116, 117]. Though socioeconomic factors may not have a direct biophysical effect, they influence mobility indirectly through psychological and sociobehavioural mediation on healthy ageing [117]. Educational disadvantage early in life can restrict occupational choice and income potential, leading to poorer living conditions and limited access to health-promoting environments, which in turn influences mobility in older adults [114]. Financial security is a vital factor in ageing experiences. Older adults with stable finances can afford essential health services, medications, balanced diets, mobility aids, tickets to recreational facilities, and social events [118]. Beltrán-Sánchez et al. [114] reported a significant influence of job type, education, and wealth on mobility in older adults, with emphasis on the negative impact of physically demanding jobs, even after controlling for age and sex. Thus, mobility disparities observed in older age partly represent the endpoint of a socially stratified trajectory rather than a purely biophysical process. The life course and SDH discourses [24, 25, 103] suggest that conducive sociopolitical environment, including formal education, employment opportunities, and social insurance, will enable healthy ageing, including mobility in later life.

There was insufficient evidence regarding the influence of marital status, area of residence, homeownership, and social status, with fewer than four studies for each factor and inconsistent results, preventing a precise conclusion. For example, a study reported mixed results on the association between marital status and physical activities [100], while related literature reviews have shown the benefits of marriage for older adults, including companionship, co-caregiving, higher household incomes, life satisfaction, social participation, and better health [119]. Specifically, studies reported that married older adults had lesser mobility difficulties compared to unmarried [120, 121] and people who lost their spouses, especially widows [104, 122, 123]. Married individuals have more household income, access to care, housing, and social support, which can help them maintain physical activity and engagement in daily activities [120]. Widowed older adults may be encouraged to get a partner, cohabit, or coreside for companionship [120, 122] or actively engage in community-based social organisations for older adults as a way of maintaining mobility and healthy ageing. Government and private organisations may offer shared living and social services at subsidised fees for older adults with lower incomes.

Home type and ownership affect mobility; homeowners and co-residents in shared living are generally more active due to home chores, while institutionalised older adults tend to be less mobile [92, 124, 125]. Rural and urban areas offer different benefits; rural areas provide natural environments (vegetation, water bodies, social and food security, quality air, and traffic safety) conducive to healthy ageing, while urban areas offer better access to healthcare and recreational services [55]. These contextual variations again reflect the SDH framework, in which environmental and infrastructural factors shape health opportunities. Government ageing policies should prioritise housing security and amenities to support healthy ageing in the community [126].

Our review made obvious the paucity of studies on the association between performance-based mobility outcomes and religious participation. Few studies suggest that religious older adults have better mobility outcomes [127, 128], but none met the inclusion criteria for this review. Similarly, a systematic review of six studies [129] suggests a positive relationship between religiosity and better functional capacity in older adults, possibly due to social support, coping mechanisms, and a sense of purpose provided by religious beliefs and practices.

### Implications for future studies

This study reveals significant gaps in the literature, including the paucity of studies with a specific objective of examining the sociodemographic factors in mobility trajectory across the life course. Standardising sociodemographic categories in observational studies, such as consistent categorisation of education by years of schooling or levels of educational attainment, would facilitate cross-study comparisons and meta-synthesis of research findings. Reporting both bivariate and multivariate outcomes would allow a better understanding of the crude and adjusted effects of the factors. Longitudinal studies are encouraged, where feasible, to establish modifiable sociodemographic determinants of performance-based mobility outcomes. Longitudinal studies can also facilitate analysis of sociodemographic interactions in mobility decline trajectory and identify the effect of changes in sociodemographic statuses, such as moving from widowed to married and vice versa. Future research could incorporate intersectionality within the SDH framework, considering that age, race, gender, education, occupation, income, residential area, housing type, and social status may collectively shape individual health trajectories through complex interactions.

### Policy and clinical implications

Ageing is a universal process, making older adults’ health a societal and policy priority. Addressing ageism and structural inequities requires a cultural reorientation that embeds social justice within health policy [101]. Equity-driven approaches must explicitly address sociodemographic disparities to ensure fair distribution of national resources, infrastructure, and welfare supports that mitigate mobility decline and assist those with functional limitations [28]. The World Health Organization (WHO) SDH framework underscores that equitable access to education, economic opportunities, social services, housing, preventive care, and culturally responsive mobility interventions is critical to narrowing these inequities [24].

Consistent links between lower education, female gender, and racialised status with poorer mobility outcomes highlight the need for targeted screening and intervention [16, 33, 92]. Health systems should integrate risk-stratified mobility assessments such as the TUG into routine check-ups for older adults with less than secondary education, women over 60 years, and individuals from Indigenous, Black, and other marginalised groups. Early identification of functional decline in these subpopulations enables timely referral to rehabilitation and wellness programmes [104].

Community-based interventions should dismantle structural barriers to social participation, in line with the United Nations and WHO Decade of Healthy Ageing (2021–2030) initiative [130]. In this light, federal, provincial, and municipal governments can fund culturally tailored social services and activity centres, particularly in low-income areas, offering social engagement programmes, job opportunities, transport vouchers, food pantries, and free intergenerational fitness sessions to promote healthy ageing. Partnering with faith-based organisations, settlement agencies, and older adults’ associations enhances outreach, trust, and cultural resonance.

The United Nations assesses how Member States incorporate ageing into social development and recommends policy priorities such as strengthening care systems and integrating social prescribing in primary health care [131]. Clinicians working in areas with significant inequity should have defined referral processes for subsidised social services, fitness programmes, mobility devices and technology, and peer-led groups, based on cultural preferences.

Long-term policies must address life-course determinants of mobility, as outlined in the WHO Age-Friendly Cities and Communities framework [132]. Regional development strategies should foster accessibility, engagement, autonomy, and opportunities for lifelong learning. Investments in adult education, vocational training, and flexible employment can reduce cumulative socioeconomic disadvantage. Integrating clinical screening, community programmes, and socioeducational reforms provides a coherent pathway to equitable healthy ageing and sustained functional independence among older adults.

### Limitations

The strength of our study lies in its methodological rigour. However, its limitations include the restriction to English-language articles. Further limitations arise from our focus on older adults with non-specific disease profiles. Although this approach reduced potential confounding, it limits the generalisability of our findings to apparently healthy older adults. In addition, while some multivariate models included comorbidity, the construct was inconsistently defined and typically operationalised as a dichotomous indicator or simple count rather than as disease-specific conditions. This lack of specificity restricted our ability to meaningfully incorporate comorbidity into the analysis. For these reasons, and because covariate adjustment varied widely across studies, we excluded multivariate coefficients from the meta-analysis to avoid undermining comparability and introducing bias into pooled estimates.

Consequently, only bivariate coefficients were synthesised to maintain analytical consistency, albeit at the expense of accounting for potential confounding. However, the paucity of studies and variations in mobility outcomes and statistical analyses prevented a meta-analysis on marital status, education, income, occupation, homeownership, religion, social status, area of residence, and race. For the meta-analyses, we aggregated correlation coefficients, which do not imply causality [47]. The HGS-based meta-analysis (Figs. 5 and 7) should be interpreted with caution, as variations in the walking paths employed (e.g., 2.4 m, 3.7 m, 4 m) contributed to high heterogeneity (*I²*) values. Additional caution is warranted when interpreting publication bias estimates for the meta-analyses presented in Figs. 6 and 7, since such estimates could be imprecise when fewer than ten studies are available [99].

Finally, although the review spans the entire period since MEDLINE’s inception in 1947 and provides a comprehensive historical synthesis, the final search was conducted on 27 November 2023. As a result, the most recent publications may not have been captured. However, checks of publicly available sources did not identify new evidence that would be expected to alter our conclusions.

## Conclusion

This review elucidates the multifaceted determinants of mobility limitation in older adults, highlighting the significant roles of age, gender, race, and education. While biological ageing inherently affects mobility, social factors like age, gender roles, racial identity, and socioeconomic disparities could influence mobility trajectories. The limited research on the impact of marital status, homeownership, religion, income, occupation, social status, and area of residence underscores the need for comprehensive studies that consider the broad spectrum of sociodemographic factors. Addressing these gaps will enrich our understanding of the sociodemographic influences on mobility in older adults and guide the development of targeted interventions and policies to support active ageing. 

## Supplementary Information

Supplementary Material 1.

## Data Availability

The datasets used and/or analysed during the current study are available from the corresponding author on reasonable request.

## Abbreviations

10MWT: Ten-metre Walk Test
6MinWT: Six-Minute Walk Test
CINAHL: Cumulative Index to Nursing and Allied Health Literature
CMA: Comprehensive Meta-Analysis
EMBASE: Excerpta Medica Database
HGS: Habitual Gait Speed
JBI: Joanna Briggs Institute
MEDLINE: Medical Literature Analysis and Retrieval System Online
MeSH: Medical Subject Headings
MOOSE: Meta-analysis of Observational Studies in Epidemiology
PRISMA: Preferred Reporting Items for Systematic Review and Meta-Analysis
PROSPERO: International Prospective Register of Systematic Reviews
ROB: Risk of Bias Assessment
SDH: Social Determinants of Health
SPPB: Short Physical Performance Battery
TUG: Timed Up and Go
UG: 8-Foot Up-and-Go
WHO: World Health Organisation
