# Supplementary material for: Association between sociodemographic factors and mobility among older adults: a systematic review and meta-analysis
Source: BMC Geriatr. 2026 Jan 16;26:208. doi: 10.1186/s12877-026-06984-z (PMC12903305; doi:10.1186/s12877-026-06984-z)
Supplement: Supplementary file 1 — Supplementary Material 1. [file 12877_2026_6984_MOESM1_ESM.docx]

**Supplementary File 1** PRISMA 2020 checklist, Page et al. (2021).

| **Section and Topic** | **Item #** | **Checklist item** | **Page** |
| --- | --- | --- | --- |
| **TITLE** | | |  |
| Title | 1 | Identify the report as a systematic review. | 1 |
| **ABSTRACT** | | |  |
| Abstract | 2 | See the PRISMA 2020 for Abstracts checklist. | 2 |
| **INTRODUCTION** | | |  |
| Rationale | 3 | Describe the rationale for the review in the context of existing knowledge. | 3-4 |
| Objectives | 4 | Provide an explicit statement of the objective(s) or question(s) the review addresses. | 5 |
| **METHODS** | | |  |
| Eligibility criteria | 5 | Specify the inclusion and exclusion criteria for the review and how studies were grouped for the syntheses. | 6 |
| Information sources | 6 | Specify all databases, registers, websites, organisations, reference lists and other sources searched or consulted to identify studies. Specify the date when each source was last searched or consulted. | 7 |
| Search strategy | 7 | Present the full search strategies for all databases, registers and websites, including any filters and limits used. | 7 |
| Selection process | 8 | Specify the methods used to decide whether a study met the inclusion criteria of the review, including how many reviewers screened each record and each report retrieved, whether they worked independently, and if applicable, details of automation tools used in the process. | 8 |
| Data collection process | 9 | Specify the methods used to collect data from reports, including how many reviewers collected data from each report, whether they worked independently, any processes for obtaining or confirming data from study investigators, and if applicable, details of automation tools used in the process. | 8 |
| Data items | 10a | List and define all outcomes for which data were sought. Specify whether all results that were compatible with each outcome domain in each study were sought (e.g. for all measures, time points, analyses), and if not, the methods used to decide which results to collect. | 8 |
|  | 10b | List and define all other variables for which data were sought (e.g. participant and intervention characteristics, funding sources). Describe any assumptions made about any missing or unclear information. | 8 |
| Study risk of bias assessment | 11 | Specify the methods used to assess risk of bias in the included studies, including details of the tool(s) used, how many reviewers assessed each study and whether they worked independently, and if applicable, details of automation tools used in the process. | 8 |
| Effect measures | 12 | Specify for each outcome the effect measure(s) (e.g. risk ratio, mean difference) used in the synthesis or presentation of results. | 9 |
| Synthesis methods | 13a | Describe the processes used to decide which studies were eligible for each synthesis (e.g. tabulating the study intervention characteristics and comparing against the planned groups for each synthesis (item #5)). | 9 |
|  | 13b | Describe any methods required to prepare the data for presentation or synthesis, such as handling of missing summary statistics, or data conversions. | 9 |
|  | 13c | Describe any methods used to tabulate or visually display results of individual studies and syntheses. | 9 |
|  | 13d | Describe any methods used to synthesise results and provide a rationale for the choice(s). If meta-analysis was performed, describe the model(s), method(s) to identify the presence and extent of statistical heterogeneity, and software package(s) used. | 9 |
|  | 13e | Describe any methods used to explore possible causes of heterogeneity among study results (e.g. subgroup analysis, meta-regression). | 10 |
|  | 13f | Describe any sensitivity analyses conducted to assess robustness of the synthesised results. | 10 |
| Reporting bias assessment | 14 | Describe any methods used to assess risk of bias due to missing results in a synthesis (arising from reporting biases). | 10 |
| Certainty assessment | 15 | Describe any methods used to assess certainty (or confidence) in the body of evidence for an outcome. | NA |
| **RESULTS** | | |  |
| Study selection | 16a | Describe the results of the search and selection process, from the number of records identified in the search to the number of studies included in the review, ideally using a flow diagram. | 10 |
|  | 16b | Cite studies that might appear to meet the inclusion criteria, but which were excluded, and explain why they were excluded. | 10 |
| Study characteristics | 17 | Cite each included study and present its characteristics. | 10-11 |
| Risk of bias in studies | 18 | Present assessments of risk of bias for each included study. | 11 |
| Results of individual studies | 19 | For all outcomes, present, for each study: (a) summary statistics for each group (where appropriate) and (b) an effect estimate and its precision (e.g. confidence/credible interval), ideally using structured tables or plots. | 11-18 |
| Results of syntheses | 20a | For each synthesis, briefly summarise the characteristics and risk of bias among contributing studies. | 11-18 |
|  | 20b | Present results of all statistical syntheses conducted. If meta-analysis was done, present for each the summary estimate and its precision (e.g. confidence/credible interval) and measures of statistical heterogeneity. If comparing groups, describe the direction of the effect. | 11-18 |
|  | 20c | Present results of all investigations of possible causes of heterogeneity among study results. | 11-18 |
|  | 20d | Present results of all sensitivity analyses conducted to assess the robustness of the synthesised results. | 11-18 |
| Reporting biases | 21 | Present assessments of risk of bias due to missing results (arising from reporting biases) for each synthesis assessed. | 11-18 |
| Certainty of evidence | 22 | Present assessments of certainty (or confidence) in the body of evidence for each outcome assessed. | NA |
| **DISCUSSION** | | |  |
| Discussion | 23a | Provide a general interpretation of the results in the context of other evidence. | 19-24 |
|  | 23b | Discuss any limitations of the evidence included in the review. | 26-27 |
|  | 23c | Discuss any limitations of the review processes used. | 26-27 |
|  | 23d | Discuss implications of the results for practice, policy, and future research. | 24-26 |
| **OTHER INFORMATION** | | |  |
| Registration and protocol | 24a | Provide registration information for the review, including register name and registration number, or state that the review was not registered. | 5 |
|  | 24b | Indicate where the review protocol can be accessed, or state that a protocol was not prepared. | 5 |
|  | 24c | Describe and explain any amendments to information provided at registration or in the protocol. | 5 |
| Support | 25 | Describe sources of financial or non-financial support for the review, and the role of the funders or sponsors in the review. | 29 |
| Competing interests | 26 | Declare any competing interests of review authors. | 29 |
| Availability of data, code and other materials | 27 | Report which of the following are publicly available and where they can be found: template data collection forms; data extracted from included studies; data used for all analyses; analytic code; any other materials used in the review. | 28 |

**Supplementary File 2** Search Strategies for the Six Databases

|  | **CINAHL Database Search Strategy** | |  |
| --- | --- | --- | --- |
| **ID#** | **Search term** | **Search options** | **Results** |
| S25 | S3 AND S8 AND S23 | **Expanders**- Apply equivalent subjects  **Narrow by Language**:- English  **Search modes**- Boolean/Phrase | 3,000 |
| S24 | S3 AND S8 AND S23 | **Expanders**- Apply equivalent subjects  **Search modes**- Boolean/Phrase | 3,085 |
| S23 | S9 OR S10 OR S11 OR S12 OR S13 OR S14 OR S15 OR S16 OR S17 OR S18 OR S19 OR S20 OR S21 OR S22 | **Expanders**- Apply equivalent subjects  **Search modes**- Boolean/Phrase | 1,328,779 |
| S22 | (employ* or unemploy* or occupation*) | **Expanders**- Apply equivalent subjects  **Search modes**- Boolean/Phrase | 408,804 |
| S21 | (MH "Employment+") | **Expanders**- Apply equivalent subjects  **Search modes**- Boolean/Phrase | 54,406 |
| S20 | (((social or socioeconomic or economic) N3 (status* or class*)) or income or poverty) | **Expanders**- Apply equivalent subjects  **Search modes**- Boolean/Phrase | 160,178 |
| S19 | (MH "Income+") | **Expanders**- Apply equivalent subjects  **Search modes**- Boolean/Phrase | 60,761 |
| S18 | (gender* or sex) | **Expanders**- Apply equivalent subjects  **Search modes**- Boolean/Phrase | 420,076 |
| S17 | (MH "Sex Factors") OR (MH "Gender Identity+") | **Expanders**- Apply equivalent subjects  **Search modes**- Boolean/Phrase | 143,940 |
| S16 | (ethnic* or race or racial" or immigrant*) | **Expanders**- Apply equivalent subjects  **Search modes**- Boolean/Phrase | 176,575 |
| S15 | (MH "Ethnic Groups+") | **Expanders**- Apply equivalent subjects  **Search modes**- Boolean/Phrase | 171,096 |
| S14 | (health N3 (determinant* or equity or equities or inequit* or inequal* or equality or equalities or disparit*)) | **Expanders**- Apply equivalent subjects  **Search modes**- Boolean/Phrase | 54,329 |
| S13 | (MH "Health inequities") | **Expanders**- Apply equivalent subjects  **Search modes**- Boolean/Phrase | 1,616 |
| S12 | (MH "Healthcare Disparities") | **Expanders**- Apply equivalent subjects  **Search modes**- Boolean/Phrase | 20,209 |
| S11 | ((social or socioeconomic or economic or population*) N3 (determinant* or factor* or risk* or equity or equities or inequit* or inequal* or equality or equalities or disparit*)) | **Expanders**- Apply equivalent subjects  **Search modes**- Boolean/Phrase | 184,910 |
| S10 | (MH "Socioeconomic Factors+") | **Expanders**- Apply equivalent subjects  **Search modes**- Boolean/Phrase | 60,134 |
| S9 | (MH "Social Determinants of Health") | **Expanders**- Apply equivalent subjects  **Search modes**- Boolean/Phrase | 11,754 |
| S8 | S4 OR S5 OR S6 OR S7 | **Expanders**- Apply equivalent subjects  **Search modes**- Boolean/Phrase | 21,822 |
| S7 | (mobilit” N5 limit") | **Expanders**- Apply equivalent subjects  **Search modes**- Boolean/Phrase | 2,527 |
| S6 | ((walk* or gait or ambulat* or locomot*) N5 (speed” or pace* or difficult*)) | **Expanders**- Apply equivalent subjects  **Search modes**- Boolean/Phrase | 12,920 |
| S5 | (MH "Walking Speed") | **Expanders**- Apply equivalent subjects  **Search modes**- Boolean/Phrase | 2,228 |
| S4 | (MH "Physical Mobility") | **Expanders**- Apply equivalent subjects  **Search modes**- Boolean/Phrase | 7,539 |
| S3 | S1 OR S2 | **Expanders**- Apply equivalent subjects  **Search modes**- Boolean/Phrase | 1,068,746 |
| S2 | (elderly or senior or seniors or "older adult" or "older adults" or geriatric or geriatrics or retir* or "old people” or older-age or “old age" or "older people") | **Expanders**- Apply equivalent subjects  **Search modes**- Boolean/Phrase | 340,424 |
| S1 | (MH "Aged+") | **Expanders**- Apply equivalent subjects  **Search modes**- Boolean/Phrase | 957,717 |

|  | **SPORTSDiscus Database Search Strategy** | |  |
| --- | --- | --- | --- |
| **ID#** | **Search term** | **Search options** | **Results** |
| S18 | S3 AND S7 AND S16 | **Expanders**- Apply equivalent subjects  **Narrow by Language**:- English  **Search modes**- Boolean/Phrase | 346 |
| S17 | S3 AND S7 AND S16 | **Expanders**- Apply equivalent subjects  **Search modes**- Boolean/Phrase | 364 |
| S16 | S8 OR S9 OR S10 OR S11 OR S12 OR S13 OR S14 OR S15 | **Expanders**- Apply equivalent subjects  **Search modes**- Boolean/Phrase | 284,297 |
| S15 | (employ* or unemploy* or occupation*) | **Expanders**- Apply equivalent subjects  **Search modes**- Boolean/Phrase | 96,564 |
| S14 | (((social or socioeconomic or economic) N3 (status* or class*)) or income or poverty) | **Expanders**- Apply equivalent subjects  **Search modes**- Boolean/Phrase | 22,684 |
| S13 | DE “HEALTH & income” | **Expanders**- Apply equivalent subjects  **Search modes**- Boolean/Phrase | 8 |
| S12 | (gender* or sex) | **Expanders**- Apply equivalent subjects  **Search modes**- Boolean/Phrase | 78,394 |
| S11 | DE “GENDER” OR DE “GENDER differences (Sociology)” OR DE “GENDER identity” OR DE “GENDER role” | **Expanders**- Apply equivalent subjects  **Search modes**- Boolean/Phrase | 3,505 |
| S10 | (ethnic* or race or racial" or immigrant*) | **Expanders**- Apply equivalent subjects  **Search modes**- Boolean/Phrase | 102,306 |
| S9 | (health N3 (determinant* or equity or equities or inequit* or inequal* or equality or equalities or disparit*)) | **Expanders**- Apply equivalent subjects  **Search modes**- Boolean/Phrase | 57,36 |
| S8 | ((social or socioeconomic or economic or population*) N3 (determinant* or factor* or risk* or equity or equities or inequit* or inequal* or equality or equalities or disparit*)) | **Expanders**- Apply equivalent subjects  **Search modes**- Boolean/Phrase | 17,738 |
| S7 | S4 OR S5 OR S6 | **Expanders**- Apply equivalent subjects  **Search modes**- Boolean/Phrase | 8,393 |
| S6 | (mobilit* N5 limit*) | **Expanders**- Apply equivalent subjects  **Search modes**- Boolean/Phrase | 775 |
| S5 | ((walk* or gait or ambulat* or locomot*) N5 (speed* or pace* or difficult*)) | **Expanders**- Apply equivalent subjects  **Search modes**- Boolean/Phrase | 7,722 |
| S4 | DE "WALKING speed" | **Expanders**- Apply equivalent subjects  **Search modes**- Boolean/Phrase | 1,199 |
| S3 | S1 OR S2 | **Expanders**- Apply equivalent subjects  **Search modes**- Boolean/Phrase | 79,399 |
| S2 | (elderly or senior or seniors or "older adult" or "older adults" or geriatric or geriatrics or retir* or "old people” or older-age or “old age" or "older people") | **Expanders**- Apply equivalent subjects  **Search modes**- Boolean/Phrase | 79,399 |
| S1 | DE “OLDER people” | **Expanders**- Apply equivalent subjects  **Search modes**- Boolean/Phrase | 12,625 |

|  | **MEDLINE Database Search Strategy** | |
| --- | --- | --- |
| **ID#** | **Search term** | **Results** |
| 1 | exp Aged/ [MeSH] | 3,470,331 |
| 2 | (elderly or senior or seniors or "older adult" or "older adults" or geriatric or geriatrics or "old people" or older-age or "old age" or "older people").ti,ab. | 514,296 |
| 3 | or/1-2 | 3,600,848 |
| 4 | Mobility Limitation/ [MeSH] | 5,291 |
| 5 | Walking Speed/ [MeSH] | 2,784 |
| 6 | ((walk* or gait* or ambulat* or locomot*) adj5 (speed* or pace* or difficult*)).ti,ab. | 22,834 |
| 7 | (mobilit* adj5 limit*).ti,ab. | 4,649 |
| 8 | or/4-7 | 31,297 |
| 9 | Social Determinants of Health/ [MeSH] | 6,775 |
| 10 | exp Socioeconomic Factors/ [MeSH] | 515,480 |
| 11 | ((social or socioeconomic or economic or population*) adj3 (determinant* or factor* or risk* or equity or equities or inequit* or inequal* or equality or equalities or disparit*)).ti,ab. | 148,113 |
| 12 | (health adj3 (determinant* or equity or equities or inequit* or inequal* or equality or equalities or disparit*)).ti,ab. | 22,466 |
| 13 | "determinants of health".ti,ab. | 20,350 |
| 14 | exp Ethnic Groups/ [MeSH] | 52,117 |
| 15 | exp Continental Population Groups/ [MeSH] | 108,580 |
| 16 | (ethnic* or race or racial* or immigrant*).ti,ab. | 105,716 |
| 17 | exp Gender Identity/ [MeSH] | 285,112 |
| 18 | Sex/ [MeSH] | 24,392 |
| 19 | (gender* or sex).ti,ab. | 907,836 |
| 20 | exp Income/ [MeSH] | 71,371 |
| 21 | (((social or socioeconomic or economic) adj3 (status* or class*)) or income or poverty).ti,ab. | 235,880 |
| 22 | exp Employment/ [MeSH] | 100,850 |
| 23 | (employ* or unemploy* or occupation*).ti,ab. | 752,436 |
| 24 | or/9-23 | 2,413,737 |
| 25 | 3 and 8 and 24 | 3,682 |
| 26 | limit 25 to English language | 3,577 |

|  | **EMBASE Database Search Strategy** | |
| --- | --- | --- |
| **ID#** | **Search term** | **Results** |
| 1 | exp aged/ | 3,680,976 |
| 2 | (elderly or senior or seniors or "older adult" or "older adults" or geriatric or geriatrics or retir* or "old people" or older-age or "old age" or "older people").ti,ab. | 820,149 |
| 3 | 1 or 2 | 3,967,969 |
| 4 | walking difficulty/ | 16,648 |
| 5 | walking speed/ | 24,094 |
| 6 | ((walk* or gait* or ambulat* or locomot*) adj5 (speed* or pace* or difficult*)).ti,ab. | 40,043 |
| 7 | (mobilit* adj5 limit*).ti,ab. | 7,976 |
| 8 | or/4-7 | 66,324 |
| 9 | “social determinants of health”/ | 20,707 |
| 10 | exp socioeconomics/ | 1,366,344 |
| 11 | ((social or socioeconomic or economic or population*) adj3 (determinant* or factor* or risk* or equity or equities or inequit* or inequal* or equality or equalities or disparit*)).ti,ab. | 228,490 |
| 12 | health care disparity/ | 22,868 |
| 13 | health disparity/ | 36,163 |
| 14 | (health adj3 (determinant* or equity or equities or inequit* or inequal* or equality or equalities or disparit*)).ti,ab. | 74,196 |
| 15 | ethnicity/ | 121,764 |
| 16 | race/ | 87,522 |
| 17 | (ethnic* or race or racial* or immigrant*).ti,ab. | 472,856 |
| 18 | exp gender identity/ | 22,606 |
| 19 | (gender* or sex).ti,ab. | 1,552,091 |
| 20 | exp income/ | 134,884 |
| 21 | (((social or socioeconomic or economic) adj3 (status* or class*)) or income or poverty).ti,ab. | 347,134 |
| 22 | exp employment/ | 128,428 |
| 23 | (employ* or unemploy* or occupation*).ti,ab. | 1,156,995 |
| 24 | or/9-23 | 4,319,714 |
| 25 | 3 and 8 and 24 | 6,393 |
| 26 | limit 25 to english language | 6,268 |

| **Web of Science Database Search Strategy** | | |
| --- | --- | --- |
| **ID#** | **Search term** | **Results** |
| 13 | #1 AND #4 AND #11 AND English (Languages) | 2,832 |
| 12 | #1 AND #4 AND #11 | 2,883 |
| 11 | #5 OR #6 OR #7 OR #8 OR #9 OR #10 | 4,999,925 |
| 10 | TS=((employ* or unemploy* or occupation*)) | 2,437,551 |
| 9 | TS=((((social or socioeconomic or economic) NEAR/3 (status* or class*)) or income or poverty)) | 605,445 |
| 8 | TS=((gender* or sex)) | 1,550,130 |
| 7 | TS=((ethnic* or race or racial* or immigrant*)) | 662,817 |
| 6 | TS=((health NEAR/3 (determinant* or equity or equities or inequit* or inequal* or equality or equalities or disparit*))) | 86,952 |
| 5 | TS=(((social or socioeconomic or economic or population*) NEAR/3 (determinat* or factor* risk* or equity or equities or inequit* or inequal* or equality or equalities or disparit*))) | 330,041 |
| 4 | #3 OR #2 | 47,433 |
| 3 | TS=((mobilit* NEAR/5 limit*)) | 13,394 |
| 2 | TS=((walk* or gait* or ambulat* or locomot*) NEAR/5 (speed* or pace* or difficult*)) | 34,679 |
| 1 | (elderly or senior or seniors or “older adult” or “older adults” or geriatric or geriatrics or retir* or “old people” or older-age or “old age” or “older people”) (Topic) | 792,179 |

|  | **AgeLine Database Search Strategy** | |  |
| --- | --- | --- | --- |
| **ID#** | **Search term** | **Search options** | **Results** |
| S20 | S3 AND S7 AND S17 | **Expanders**- Apply equivalent subjects  **Search modes**- Boolean/Phrase | 1,154 |
| S19 | S8 OR S9 OR S10 OR S11 OR S12 OR S13 OR S14 OR S15 OR S16 OR S17 OR S18 | **Expanders**- Apply equivalent subjects  **Search modes**- Boolean/Phrase | 70,408 |
| S18 | (employ* or unemploy* or occupation*) | **Expanders**- Apply equivalent subjects  **Search modes**- Boolean/Phrase | 30,588 |
| S17 | DE “Employment” OR DE “Alternative Work Patterns” OR DE “Part Time Employment” OR DE “Postretirement Work” OR DE “Reemployment” OR DE “Self Employment” | **Expanders**- Apply equivalent subjects  **Search modes**- Boolean/Phrase | 4,148 |
| S16 | (((social or socioeconomic or economic) N3 (status* or class*)) or income or poverty) | **Expanders**- Apply equivalent subjects  **Search modes**- Boolean/Phrase | 21,238 |
| S15 | DE “Income” OR DE “Family Income” OR DE “Household Income” OR DE “Lifetime Income” OR DE “Retirement Income” | **Expanders**- Apply equivalent subjects  **Search modes**- Boolean/Phrase | 3,620 |
| S14 | (gender* or sex) | **Expanders**- Apply equivalent subjects  **Search modes**- Boolean/Phrase | 20,174 |
| S13 | DE “Sex Differences” | **Expanders**- Apply equivalent subjects  **Search modes**- Boolean/Phrase | 5,439 |
| S12 | (ethnic* or race or racial" or immigrant*) | **Expanders**- Apply equivalent subjects  **Search modes**- Boolean/Phrase | 13,983 |
| S11 | DE “Racial and Ethnic Groups” OR DE “Aborigines” OR DE “Afghans” OR DE “Africans” OR DE “American Indians” OR DE “Arabs” OR DE “Armenians” OR DE “Asians” OR DE “Australians” OR DE “Azerbaijanis” OR DE “Bangladeshis” OR DE “Blacks” OR DE “Bosnians” OR DE “British” OR DE “Cambodians” OR DE “Canadians” OR DE “Caribbeans” OR DE “Chamorros” OR DE “Chinese” OR DE “Cubans” OR DE “Cypriots” OR DE “Czechs” OR DE “Dominicans” OR DE “Dutch” OR DE “Eastern Europeans” OR DE “Estonians” OR DE “Europeans” OR D … | **Expanders**- Apply equivalent subjects  **Search modes**- Boolean/Phrase | 7,860 |
| S10 | (health N3 (determinant* or equity or equities or inequit* or inequal* or equality or equalities or disparit*)) | **Expanders**- Apply equivalent subjects  **Search modes**- Boolean/Phrase | 1,884 |
| S9 | ((social or socioeconomic or economic or population*) N3 (determinant* or factor* or risk* or equity or equities or inequit* or inequal* or equality or equalities or disparit*)) | **Expanders**- Apply equivalent subjects  **Search modes**- Boolean/Phrase | 5,223 |
| S8 | DE “Socioeconomic Levels” OR DE “Income Levels” OR DE “Living Standards” OR DE “Poverty Levels” OR DE “Social Classes” | **Expanders**- Apply equivalent subjects  **Search modes**- Boolean/Phrase | 5,823 |
| S7 | S4 OR S5 OR S6 | **Expanders**- Apply equivalent subjects  **Search modes**- Boolean/Phrase | 3,794 |
| S6 | (mobilit* N5 limit*) | **Expanders**- Apply equivalent subjects  **Search modes**- Boolean/Phrase | 554 |
| S5 | ((walk* or gait or ambulat* or locomot*) N5 (speed* or pace* or difficult*)) | **Expanders**- Apply equivalent subjects  **Search modes**- Boolean/Phrase | 1,870 |
| S4 | DE "Walking" | **Expanders**- Apply equivalent subjects  **Search modes**- Boolean/Phrase | 2,247 |
| S3 | S1 OR S2 | **Expanders**- Apply equivalent subjects  **Search modes**- Boolean/Phrase | 169,569 |
| S2 | (elderly or senior or seniors or "older adult" or "older adults" or geriatric or geriatrics or retir* or "old people” or older-age or “old age" or "older people") | **Expanders**- Apply equivalent subjects  **Search modes**- Boolean/Phrase | 169,568 |
| S1 | DE “Older Adults” | **Expanders**- Apply equivalent subjects  **Search modes**- Boolean/Phrase | 133,497 |

**Supplementary File 3** Characteristics of the included studies (*n* = 57)

| **First author surname, Year &**  **Country** | | **Age range**  **[mean] years** | **Sample size** | **% Female** | **Dataset name** | **Relevant mobility tools & Outcomes** | **Title** | **Analysis type & Results** |
| --- | --- | --- | --- | --- | --- | --- | --- | --- |
| Al Snih, 2008  USA | | ≥60  [69.2] | 4,456 | 49.2 | NHANES III | 8ft UGT  Walk time (s) | Ethnic differences in physical performance in older Americans: data from the Third National Health and Nutrition Examination Survey (1988-1994) | BAS: Non-Hispanic Whites had better mobility than Mexicans and Blacks (SE = 0.07, p<0.001). MAS: younger age (β= 0.07, p<0.0001), male (β= 0.29, p<0.01), higher education (β= -0.37, p<0.1), and being Non-Hispanic White vs Black (β= 0.45, p<0.001) or vs Mexican (β= 0.39, p<001) were associated with better mobility. No statistically significant effect of marital status (β= -0.22, p>0.05). |
| Aoyagi, 2001  USA | | ≥65  [72.3] | 10,247 | 100.0 | MBJS | 6 MWT  Gait speed (m/s) | Comparison of performance-based measures among native Japanese, Japanese-Americans in Hawaii and Caucasian women in the United States, ages 65 years and over: A cross-sectional study | BAS: Usual walking speed was about 10% slower among Caucasians than native Japanese, whereas Japanese-Americans in Hawaii walked about 11% faster than native Japanese. |
| Asher, 2012  UK | | ≥65  [74.3] | 3,145 | 54.1 | HSE 2005 | 2.4 MWT  Gait speed (m/s) | Most older pedestrians are unable to cross the road in time: a cross-sectional study | BAS: younger age (OR= 6.63, p< 0.001), males (OR= 2.40, p<0.001), and higher education (OR= 5.20, p<0.001) were associated with better mobility. MAS: younger age (OR= 3.65, p<0.001) and being male (OR= 2.64, p<0.001) were associated with better mobility. |
| Barrera, 2017  Chile | | [73.0] | 86 | 100.0 | NA | TUG,  Walk time (s) | Associations between socioeconomic status, aging and functionality among older women | BAS: Illiterate or primary education participants (Median TUG score=7.8) had significantly lower mobility than secondary (M=6.4) and higher education (M=6.1) p<0.05. |
| Bendall, 1989  UK | | 65–90  [71.5] | 125 | 53.6 | NA | Sensor, 100 MWT  Gait speed (m/s) | Factors affecting walking speed of elderly people | BAS: There was a negative correlation between gait speed and age in men (r = -0.32, p<0.01) and women (r = -0.28, p<0.05). However, men had higher gait speed than women (p<0.001). |
| Binotto, 2019  Brazil | | ≥60 | 421 | 30.2 | NA | 4.6 MWT  Gait speed (m/s) | Gait speed associated factors in elderly subjects undergoing exams to obtain the driver's license Portuguese, English, Spanish | MAS: younger age (β= -0.01, p<0.001) and being a man (β= 0.07, p=0.026) were associated with better mobility when controlled for body mass index and hand grip. |
| Blanco, 2012  USA | | [77.7] | 213 | 65.3 | EAS | Sensor, 3.7 MWT  Gait speed (m/s) | Racial differences in gait velocity in an urban elderly cohort | BAS: Caucasian (β= -8.87, p<0.01) had better mobility than African Americans. There was no significant gender difference in mobility (p=0.10). MAS: Being Caucasian (β= -7.49, p=0.015) was associated with better mobility adjusted for age, gender, BMI, education, and chronic diseases. |
| Bohannon, 1996  USA | | 50–79  [64.3] | 156 | 50.6 | NA | 7.6 MWT  Gait speed (m/s) | Walking speed: Reference values and correlates for older adults | BAS: Being a woman significantly correlated with lower gait speed (r= -0.254, p<0.01). There was no significant correlation between age and gait speed (r= -0.026, p>0.05). MAS: Being a man (R= 0.362, p<0.001) was associated with better mobility, adjusted for weight and strength of hip flexion. |
| Bohannon, 2008  USA | | ≥50  [68.7] | 1,923 | 49.3 | NHANES | 8 ft WT  Gait speed (m/s) | Population representative gait speed and its determinants | BAS: There was a significant correlation between lower mobility and older age (r= -0.354, p<0.001) and being a woman (r= -0.060, p=0.025). MAS: Younger age was associated with better mobility (β = -0.015, p=0.002), adjusted for knee extension force, waist circumference, stature, and gender. |
| Boulifard, 2019  USA | | ≥65  [74.8] | 6,983 | 54.2 | HRS | 2.5 MWT  Gait speed (m/s) | Home-based gait speed assessment: Normative data and racial/ethnic correlates among older adults | BAS: younger persons (M/SD = 8.53/2.55 vs 7.12/2.32, p<0.05), male (M = 8.32/2.56 vs 7.56/2.48, p<0.05), and Caucasians (t = -12.8, p<0.001) had better mobility. MAS: younger age (B= -0.9, p<0.001), men (B= -4.44, p<0.001), higher income (B = 1.1, p<0.001), higher education (B = 0.6, p<0.001), urbanicity (B= -1.0, p=0.004) and being Caucasian (B = -9.6, p<0.001) were associated with better mobility; adjusted for urbanicity, chronic disease, and health behaviours. |
| Brunner, 2009  UK | | 50–74  [61.1] | 6,345 | 29.3 | Whitehall II study | 2.4 MWT  Gait speed (m/s) | Social inequality in walking speed in early old age in the Whitehall II study | BAS: Younger people, men, married, higher incomes, and Caucasians had better mobility (p<0.05), but there was no significant effect of social status (p>0.05). Average age- and ethnicity-adjusted walking speed was approximately 13% higher in the highest employment grade compared to the lowest. Based on the relative index of inequality (RII), the difference in walking speed across the social hierarchy was 0.15 m/s in men and 0.17 in women, corresponding to an age-related difference of 18.7 years in men and 14.9 years in women. |
| Buchner, 1996  USA | | 60–90  [75.3] | 409 | 60.0 | NA | 15.2 MWT  Gait speed (m/s) | Evidence for a non-linear relationship between leg strength and gait speed | BAS: Younger age (r =-0.49, p<0.05) and being a man (r= 0.10, p<0.05) correlated with better mobility. |
| Busch, 2015  Brazil | | ≥60 | 1,112 | 60.3 | SABE 2010 | 3 MWT  Gait speed (m/s) | Factors associated with lower gait speed among the elderly living in a developing country: a cross-sectional population-based study. | BAS: Younger people (p<0.001) and people with higher education (p<0.001) had better mobility. There was no significant association between gender (p=0.987) and race (p=0.939) with mobility. MAS: Factors associated with lower gait speed were being older (OR = 3.56, p<0.001), being illiterate (OR = 3.20, p=0.017), having difficulty in one or more IADL (OR = 2.74, p<0.001), presence of CVD (OR = 2.15, p=0.006) and being active as a protection factor (OR = 0.56, p=0.027). |
| Butler, 2009  Australia | | 75–98  [80.1] | 684 | 65.2 | NA | 6 MWT  Gait speed (m/s) | Age and gender differences in seven tests of functional mobility | BAS: Older participants performed significantly worse than the younger participants in all of the functional mobility tests (p < 0.001), with the older women performing worse than the older men in all of the tests (p < 0.05). A significant correlation was found between age and gait speed scores (r= -0.30, p<0.001). |
| Carvalho de Abreu, 2021  Brazil | | ≥60  [70.3] | 233 | 64.4 | NA | Sensor, 8 MWT  Gait speed (m/s) | Functional performance of older adults: A comparison between men and women | BAS: There was no significant gender difference in gait speed (t = 1.97, p = 0.162). |
| ChilesShaffer, 2020  USA | | ≥60  [75.5] | 1,112 | 51.8 | BLSA | 6 MWT  Gait speed (m/s) | The roles of body composition and specific strength in the relationship between race and physical performance in older adults | MAS: FOR MEN, younger age (β= -0.02, p<0.001), higher income (β= 0.06, p<0.05), and being Caucasian (β= -0.10, p<0.001) were associated with better mobility, but effects of education (β= 0.01, p>0.05) and height (β= 0.03, p>0.05) were not significant in the model.  MAS: FOR WOMEN, younger age (β= -0.01, p<0.001), height (β= 0.52, p<0.001), and being Caucasian (β= -0.14, p<0.001) were associated with better mobility, but effects of education (β= 0.02, p>0.05) and higher income (β= 0.03, p>0.05) were not significant in the model. |
| Coelho-Junior, 2021  Brazil | | 50–102  [68.0] | 2,804 | 80.7 | NA | TUG, 3 MWT  Walk time (s), Gait speed (m/s) | Age- and gender-related changes in physical function in community-dwelling Brazilian adults aged 50 to 102 years | BAS: Older age correlated with higher TUG scores/mobility decline (r = 0.30, p<0.001) in both males and females. |
| Dommershuijsen, 2022  Netherlands | | ≥50  [67.7] | 4,656 | 55.2 | Rotterdam study | Sensor, 5.8 MWT  Gait speed (m/s) | Gait speed reference values in community-dwelling older adults - cross-sectional analysis from the Rotterdam Study | BAS: For both men and women, younger age and higher education were associated with better mobility (p<0.05). However, sex did not affect gait speed after accounting for age and height. |
| Fang, 2020  China | | 60–89  [73.5] | 113 | 56.0 | NA | Sensor, 2 minWT  Gait speed (m/s) | Three-dimensional thoracic and pelvic kinematics and arm swing maximum velocity in older adults using inertial sensor system | BAS: Younger people (F= 68.903, p<0.001) had better mobility but no significant effect of gender (p=0.65) or age*gender interaction (p=0.56). |
| Fiser, 2010  USA | | 60–88  [72.5] | 49 | 49.0 | NA | SPPB, HGS  Gait speed (m/s) | Energetics of walking in elderly people: factors related to gait speed | BAS: Women had slower habitual walking speeds (1.04 ± 0.04 vs 1.21 ± 0.04 m/s, p = .006) than men. |
| *Gomes, 2023  Brazil | | ≥60  [68.0] | 476 | 65.1 | COMO VAI | TUG, 4 MWT  Walk time (s), Gait speed (m/s) | Changes in physical performance among community-dwelling older adults in six years | BAS: Being male (p = 0.023), living without a partner/separated (p = 0.035), higher education (p = 0.019), and alcohol consumption in the prior month (p = 0.045) were associated with decreased GS, while older age (p<0.001), having lower socioeconomic status (p<0.004), physical inactivity (p = 0.017), and being overweight (p = 0.007) were associated with increased TUG time. |
| Granic, 2018  USA | | ≥60  [74.0] | 577 | 72.3 | HARI | 4 MWT  Gait speed (m/s) | Factors associated with physical performance measures in a multiethnic cohort of older adults | BAS: European Americans walked faster than > African American > Hispanic, and > Afro-Caribbean (p<0.001). MAS: Younger age, being a man, and higher education were associated with better mobility (p<0.01) among the multiracial cohort. |
| Ibrahim, 2017  Malaysia | | ≥60  [68.7] | 2,084 | 51.8 | LRGS TUA | TUG  Walk time (s) | ‘Timed Up and Go’ test: age, gender and cognitive impairment stratified normative values of older adults. | BAS: Younger people (p<0.001) and men (p<0.001) had better mobility. MAS: younger age (β = 0.76, p<0.001) and being a man (β = 0.89, p<0.001) were associated with better mobility, adjusted for cognitive status. |
| *Idland, 2013  Norway | | 75–92  [79.5] | 113 | 100.0 | NA | TUG  Walk time (s) | Predictors of mobility in community-dwelling women aged 85 and older. | BAS: Younger people had less mobility decline (β =0.34, p<0.001) after nine years of follow-up; education level had no significant effect (β = -0.05, p = 0.60). MAS: Being of younger age was associated with less mobility decline (β = 0.35, p < 0.001) when adjusted for living alone, step climbing score, walking habits, general health, and BMI. |
| Iwakura, 2022  Japan | | ≥65  [72.0] | 392 | 70.4 | NA | TUG  Walk time (s) | Lower-limb muscle strength and major performance tests in community-dwelling older adults | MAS: After controlling for height and weight, younger age was associated with better TUG score/mobility (β =0.264, p<0.001), but there was no statistical gender effect (β = -0.049, p = 0.319). |
| *Jerome, 2015  USA | | 60–89  [72.3] | 362 | 51.0 | BLSA | 6 MWT  Gait speed (m/s) | Gait characteristics associated with walking speed decline in older adults: results from the Baltimore Longitudinal Study of Aging | MAS: After three years of follow-up, younger age was associated with less mobility decline (OR = 1.03 [95% CI: 1.00, 1.07); the model was adjusted for initial gait speed, sex, race, height, weight, and follow-up time. |
| Kamiya, 2019  Japan | | ≥75  [80.3] | 109 | 12.8 | NA | 10 MWT, 6 minWT  Gait speed (m/s), Walk distance (m) | The 6-Minute Walk Test: Difference in explanatory variables for performance by community-dwelling older adults and patients hospitalized for cardiac disease | BAS: Oder age had a significant negative correlation with 6minWT (r = -0.367 [95% CI: -0.519, -0.192]) and 10 MWT scores (r = -0.220 [95% CI: -0.392, -0.033]). MAS: Younger age (β = -0.270, p=0.001) was significantly associated with better mobility; the effect of being a man was not significant (β =0.210, p=0.051); the model was adjusted for gait speed, grip strength, BMI, and cognition. |
| Lin, 2021  Taiwan | | ≥65  [75.0] | 301 | 55.1 | Yilan study | 6 MWT  Gait speed (m/s) | Using hand grip strength to detect slow walking speed in older adults: the Yilan study | BAS: Younger people (p<0.001), men (p<0.001), and people with higher education (p=0.001) had better mobility. Younger age correlated with better mobility (r = -0.42, p<0.001) MAS: younger age (β = -0.23, p<0.001), men (β = -0.13, p<0.001), and higher education (β = -0.15, p<0.05) were associated with better mobility. |
| Lunar, 2019  Philippines | | ≥60  [67.6] | 180 | 60.0 | NA | 10 MWT, 6 minWT  Gait speed (m/s), Walk distance (m) | Mobility performance among community-dwelling older Filipinos who lived in urban and rural settings: A preliminary study | BAS: T-test analysis showed that urban residents had better mobility than their rural counterparts (p<0.001). |
| Makizako, 2017  Japan | | ≥65  [73.6] | 10,092 | 52.5 | NCGG-SGS | 2.4 MWT  Gait speed (m/s) | Age-dependent changes in physical performance and body composition in community-dwelling Japanese older adults | BAS: Younger age was significantly correlated with better mobility in men (r= -0.37) and women (r = -0.48), both p<0.001. MAS: Older age was associated with poor mobility, β = 0.62, 95% CI = 0.66 to 0.57 in men and β = 0.95, 95% CI= 0.99 to 0.90 in women for walking speed. |
| Mantel, 2019  USA | | ≥60  [75.2] | 60 | 68.3 | NA | Sensor, 3.7 MWT  Gait speed (m/s) | An investigation of the predictors of comfortable and fast gait speed in community-dwelling older adults | BAS: Younger people (r = -0.48, p<0.001) had better mobility, and there was no statistically significant correlation with gender (r = -0.22, p>0.05). |
| Milanović, 2013  Serbia | | 60–80  [66.5] | 1,288 | 53.9 | NA | 8ft UGT  Walk time (s) | Age-related decrease in physical activity and functional fitness among elderly men and women. | BAS: Younger people [60 to 69 years] had better mobility than the older age range 70 to 80 years, in men (-16%, p<0.05) and women (-9%, p<0.05). |
| Mohammed, 2021  India | | 65–83  [73.6] | 100 | 47.0 | NA | TUG  Gait speed (m/s) | Influence of age, gender, and body mass index on balance and mobility performance in Indian community-dwelling older people | BAS: Younger age (r = 0.81, p<0.0001) correlated with better mobility, and there was no statistically significant effect of gender (d = 0.295, p=0.14). |
| Payne, 2017  South Africa | | ≥40  [61.7] | 5,058 | 54.0 | HAALSI | 5 MWT  Gait speed (m/s) | Physical function in an aging population in rural South Africa: Findings from HAALSI and cross-national comparisons with HRS sister studies | BAS: Younger people and men had better mobility (p<0.05). MAS model 1: younger age (β = -0.0035, p<0.01), men (β = 0.032, p<0.01), and being married vs. never married (β = -0.049, p<0.01) or widowed (β = -0.026, p<0.05) were associated with better mobility, but there was no statistically significant effect of education on mobility (β = 0.019, p>0.05), when controlled for HIV and CVD status. |
| Plouvier, 2016  France | | 55–69  [61.4] | 736 | 40.1 | CONSTANCES | 3 MWT  Gait speed (m/s) | Socioeconomic disparities in gait speed and associated characteristics in early old age | MAS: Compared to managers/executives, gait speed was reduced in less skilled categories among men (OR 1.21 [0.72–2.05] for Intermediate/Tradesmen, 1.95 [0.80–4.76] for Clerks, Sale/service workers, 2.09 [1.14–3.82] for Blue collar/Craftsmen) and among women (OR 1.12 [0.55–2.28] for Intermediate/Tradesmen, 2.33 [1.09–4.97] for Clerks, 2.48 [1.18–5.24] for Sale/service workers/Blue collar/Craftsmen); adjusted for age and health centre. Among men, occupational exposure to carrying heavy loads explained a large part of socioeconomic disparities. Among women, obesity and occupational exposure to repetitive work contributed independently to the disparities. |
| Ramírez-Vélez, 2020  Columbia | | ≥60  [69.0] | 4,211 | 53.0 | SABE | SPPB, 3 MWT  Gait speed (m/s) | Normative values for the short physical performance battery (SPPB) and their association with anthropometric variables in older Colombian adults. The SABE Study, 2015 | BAS: Younger people (β= -0.280, p<0.001) and men (d = 0.391, p<0.001) had better mobility. MAS: age (β= -0.248, p<0.001) was a significant contributor to walking speed after controlling for body mass, height, BMI, calf circumference, ethnicity, socioeconomic status, and urbanicity. |
| Rikli, 1999  USA | | 60–94  [73.3] | 7,183 | 70.3 | NA | 8 ftUGT, 6 MinWT  Walk time(s), Walk distance (m) | Functional fitness normative scores for community-residing older adults, ages 60–94. | BAS: ANOVA and post hoc comparison indicated a significant main effect for age and gender, such that younger people (p<0.007) and men (p<0.0001) had better mobility. |
| Ruggero, 2013  Brazil | | 65–92  [71.4] | 385 | 64.4 | FIBRA Network | 4.6 MWT  Gait speed (m/s) | Gait speed correlates in a multiracial population of community-dwelling older adults living in Brazil: a cross-sectional population-based study | BAS: Younger people (OR 3.91, p<0.001) and people with higher education (OR 1.74, p=0.039) had better mobility, but there was no significant effect of gender and income level (OR 1.29, p<0.359). MAS: A younger age was associated with better mobility (OR 3.81, p<0.001), adjusted for physical activity level, chronic conditions, and concern of falling. |
| Schrack, 2012  USA | | 32–96  [68.1] | 420 | 48.1 | BLSA | 6 MWT  Gait speed (m/s) | The role of energetic cost in the age-related slowing of gait speed | MAS: younger age (β= -0.2017, p<0.001) and being a man (β= 1.030, p=0.02) were associated with better mobility, controlled for height, male sex by height, energy expenditure, smoking status, chronic diseases, and balance performance. |
| Seino, 2014  Japan | | ≥65  [74.0] | 4,683 | 53.7 | See list‡ | 5 and 10 MWT  Gait speed (m/s) | Reference values and age and sex differences in physical performance measures for community-dwelling older Japanese: a pooled analysis of six cohort studies | BAS: T-test showed that the younger age group and men had better mobility (p<0.001). MAS: In multiple linear regression analyses, age (b=20.40, p<0.001) and sex (b=20.09, p<0.001) were significantly associated with usual gait speed. |
| Shubert, 2006  USA | | 65–103  [80.9] | 195 | 70.0 | NA | 10 MWT  Gait speed (m/s) | Are scores on balance screening tests associated with mobility in older adults? | MAS: younger age (OR=0.92, p=0.04) was associated with better mobility, with no significant gender effect (OR=1.71, p=0.26), adjusted for 360-degree turn and tandem stance. |
| †Sialino, 2019  Netherlands | | 55–65  [60.3] | 3,469 | 50.6 | LASA | 6MWT  Gait speed (m/s) | Sex differences in physical performance by age, educational level, ethnic groups and birth cohort: The Longitudinal Aging Study Amsterdam | BAS: Mixed model analysis showed that women had a lower age- and height-adjusted gait speed (-0.03 m/s; 0.063–0.001) compared to men. MAS: Older people and women had consistently lower gait speed across different educational levels and Turkish/Moroccan ethnic groups and birth cohorts. |
| *Sialino, 2021  Netherlands | | 55–81  [66.1] | 2,407 | 50.0 | LASA | 6 MWT  Gait speed (m/s) | The sex difference in gait speed among older adults: How do sociodemographic, lifestyle, social and health factors contribute? | BAS: Men had less mobility decline compared to women after 15 to 25 years of follow-up (Md=0.076m/s, p<0.001). MAS: Having a lower educational level, living alone and having more chronic diseases, pain and depressive symptoms among women compared to men contributed to observed lower gait speed in women (P<0.05). In men, being a smoker, having lower physical activity, and having a smaller personal network size compared to women contributed to a lower gait speed among men (p<0.05). |
| Smolar, 2012  USA | | ≥60  [73.5] | 148 | 69.4 | NA | 4 MWT  Gait speed (m/s) | Gait speed in community-dwelling African-American and Afro-Caribbean older adults | BAS: European Americans had better mobility than Blacks (β= -0.179, p=0.001). MAS: Younger age (β= -0.014, p<0.001) and being Caucasian (β= -0.128, p=0.02) were associated with better mobility, but there were no significant effects of gender (β=0.062, p=0.23) and education (β=0.005, p=0.40), adjusted for HbA1c, physical activity and BMI. |
| Sprague, 2023 | Tanzania | ≥65  [80.5] | 231 | 70.1 | IDEA | 10 MWT  Gait speed (m/s) | Correlates of gait speed among older adults from 6 countries: Findings from the COSMIC collaboration | BAS: Younger people (r=-0.43, P<0.001) and people with higher education (p=0.03) had better mobility, but there was no significant gender effect (p=0.09). |
|  | Nigeria | ≥65  [76.9] | 1,122 | 51.4 | ISA | 3 and 4 MWT  Gait speed (m/s) |  | BAS: Younger people (r=-0.27, p<0.001) and men (p<0.001) had better mobility, but there was no significant education effect (p=0.65). |
|  | South Korea | ≥65  [73.3] | 491 | 55.4 | KLOSCAD | 10 MWT  Gait speed (m/s) |  | BAS: Younger people (r=-0.36, p<0.001) and people with higher education (p=0.045) had better mobility, but there was no significant gender effect (p=0.25). |
|  | Japan | ≥65  [73.6] | 1,913 | 58.1 | SGS | 5 MWT  Gait speed (m/s) |  | BAS: Younger people (r=-0.42, p<0.001), women (p<0.001), and people with higher education (p<0.001) had better mobility |
|  | Singapore | ≥65  [72.4] | 1,698 | 59.1 | SLAS-II | 6 MWT  Gait speed (m/s) |  | BAS: Younger people (r=-0.32, p<0.001), men (p<0.001), and people with higher education (p<0.001) had better mobility. |
|  | Australia | ≥65  [78.8] | 995 | 55.2 | Sydney MAS | 6 MWT  Gait speed (m/s) |  | BAS: Younger people (r=-0.32, p<0.001), men (p=0.009), and people with higher education (p<0.001) had better mobility. |
| Staples, 2020  USA | | ≥60  [76.0] | 111 | 78.4 | NA | TUG, 10 MWT  Walk time (s), Gait speed (m/s) | Examination of the correlation between physical and psychological measures in community-dwelling older adults | BAS for TUG: Younger people had better mobility (r=0.413, p<0.01). MAS for TUG: A younger age (β=0.285, p=0.002) was significantly associated with better mobility, adjusted for grip strength and depression. BAS for 10MWT: Younger people had better mobility (r=-0.266, p<0.01). MAS for TUG: A younger age (β=-0.235, p=0.010) and lower level of education (β=0.194, p=0.027) were significantly associated with better mobility, adjusted for grip strength and depression. |
| Tanaka, 2022  Japan | | ≥65  [75.1] | 387 | 57.6 | NA | Motion sensor  Gait speed (m/s) | Effects of age and gender on spatiotemporal and kinematic gait parameters in older adults | BAS: Age group effects were found in the walking speed (F=14.165, p<0.001, η2 = 0.131), such that the younger age group had better mobility but no significant gender differences. |
| Tangen, 2020  Norway | | ≥70 [  74.0] | 105 | 45.7 | NA | TUG, 4 MWT  Walk time (s), Gait speed (m/s) | Measuring physical performance in highly active older adults: associations with age and gender? | BAS: Higher age was correlated with lower mobility in men (TUG: r=0.19, p>0.05, 4MWT: r=-0.27, p<0.05) and women (TUG: r=0.48, p<0.001, 4MWT: r=-0.40, p<0.001). MAS: After controlling for age, the effect of gender was not significant (p=0.292) for 4MWT and (p=0.075) for TUG. |
| Thaweewannakij, 2013  Thailand | | ≥60 | 1,030 | 68.9 | NA | TUG, 10 MWT, 6 minWT  Walk time (s), Gait speed (m/s), Walk distance (m) | Reference values of physical performance in Thai elderly people who are functioning well and dwelling in the community | BAS: The findings demonstrated that the mobility of male participants was significantly better than that of female participants in every age decade (P<0.05). MAS: The findings indicated significant age-related functional decline for both male (P<0.05) and female (P<001) participants in all mobility tests, adjusted for weight and height. |
| Thompson, 1995  USA | | 65–79  [71.5] | 175 | 68.6 | NA | TUG  Walk time (s) | Performance of community dwelling elderly on the timed up and go test | BAS: MANOVA result showed that men had better mobility than women (F=10.21, p<0.01), but age had no significant effect (F=0.50, p>0.05). |
| †Thorpe, 2011  USA | | 70–79  [73.4] | 2,969 | 51.3 | Health ABC | 6 MWT  Gait speed (m/s) | Race, socioeconomic resources, and late-life mobility and decline: findings from the Health, Aging, and Body Composition study | BAS: T-test showed that Caucasians had less mobility decline after five years of follow-up (p<0.001). MAS: Gender disaggregate logistic regression models showed that younger age, higher income, higher education, homeownership, and being Caucasian were associated with less mobility decline (p<0.05). |
| *Vasunilashorn, 2009  Italy | | ≥65  [71.6] | 542 | 51.5 | InCHIANTI | 400 MWT  Gait speed (m/s) | Use of the Short Physical Performance Battery Score to predict loss of ability to walk 400 meters: analysis from the InCHIANTI study | MAS: A younger age was associated with a higher tendency for completing 400MWT (OR=1.10, p<0.01), but there were no significant effects of gender (OR=1.42, p=0.30) and education (OR=0.98, p=0.72), adjusted for SPPB, BMI, cognition, and number of chronic diseases. |
| Watson, 2010  USA | | 70–79  [75.2] | 909 | 50.6 | ABC Cognitive Vitality Substudy | 20 MWT  Gait speed (m/s) | Executive function, memory, and gait speed decline in well-functioning older adults | BAS: In baseline, younger people (p<0.001), men (p<0.001), people with higher education (p<0.001), and Caucasians (p<0.001) had better mobility. |
| Welmer, 2013  Sweden | | ≥60  [74.2] | 3,212 | 63.8 | SNSAC | 2.4 and 6 MWT  Gait speed (m/s) | Education-related differences in physical performance after age 60: A cross-sectional study assessing variation by age, gender and occupation. | BAS: Lower gait speed was found in older participants, in women, in manual workers, and in people with lower levels of education (p<0.05). MAS: Multivariate regression showed higher education attainment was associated with better mobility (p<0.001), controlling for age, gender, chronic diseases and lifestyle-related variables. |
| Wheaton, 2016 | USA | 55–85  [66.5] | 14,125 | 55.0 | HRS | 2.5 MWT  Gait speed (m/s) | Female disability disadvantage: A global perspective on sex differences in physical function and disability. | MAS: Being a man was associated with better mobility (B= -0.07, p<0.001), adjusted for age, education, and marital status. |
|  | Taiwan | 55–85  [66.4] | 1,051 | 48.0 | SEBAS | 3 MWT  Gait speed (m/s) |  | MAS: Being a man was associated with better mobility (B= -0.13, p<0.01), adjusted for age, education, and marital status. |
|  | China | 55–85  [66.4] | 7,438 | 57.0 | CHARLS | 3 MWT  Gait speed (m/s) |  | MAS: Being a man was associated with better mobility (B= -0.03, p<0.05), adjusted for age, education, and marital status. |
|  | Bolivia | 55–85  [65.5] | 449 | 47.0 | THLHP | 2.5 MWT  Gait speed (m/s) |  | MAS: Being a man was associated with better mobility (B= -0.05, p<0.01), adjusted for age. |
| Wu, 2021  China | | 60–80  [65.8] | 211 | 70.1 | NA | 6 MWT  Gait speed (m/s) | Associations between functional fitness and walking speed in older adults | MAS: A younger age was associated with faster usual walking speed (B= -0.012, p<0.001), adjusted for 8ft UG, 2-min step, chair sit-and-reach test, and gender. |
| Yaoxin, 2022  China | | ≥65  [72.9] | 595 | 59.2 | NA | TUG  Walk time (s) | Mediating effect of lower extremity muscle strength on the relationship between mobility and cognitive function in Chinese older adults: A cross-sectional study | BAS: Younger people (F = 54.7, p<0.001) and people with higher education (F = 14.0, p<0.001) had better mobility, but there was no significant effect of gender (t = -0.32, p=0.767) or job type (F = 2.38, p=0.09). |

**Dataset:** BLSA: Baltimore Longitudinal Study of Aging. CHARLS: China Health and Retirement Longitudinal Study. COMO VAI: Consórcio de Mestrado Orientado para Valorização da Atenção ao Idoso. CONSTANCES: Cohorte des consultants des Centres d'examens de santé. EAS: Einstein Aging Study. FIBRA Network Study: Frailty among Brazilian Older Adults. HAALSI: Health and Aging in Africa: A Longitudinal Study of an INDEPTH Community in South Africa. HARI: Florida Atlantic University Healthy Aging Research Initiative. ‡HATOYAMA: Hatoyama Cohort Study. Health ABC: Health, Aging, and Body Composition Study. HRS: Health and Retirement Study. HSE-2005: Health Survey for England 2005. IDEA: Identification and Intervention for Dementia in Elderly. InCHIANTI: Invecchiare in Chianti study. ISA: Ibadan Study of Aging. ‡ITABASHI-02: Itabashi Cohort Study 2002. ‡ITABASHI-11: Itabashi Cohort Study 2011. KLOSCAD: Korean Longitudinal Study on Cognitive Aging and Dementia. ‡KUSATSU Kusatsu Longitudinal Study. LRGS TUA: Longitudinal Study on Neuroprotective Model for Healthy Longevity. LASA: Longitudinal Aging Study Amsterdam. MBJS: Mitsugi Bone and Joint Study. ‡NANGAI: Nangai Cohort Study. NCGG-SGS: National Center for Geriatrics and Gerontology-Study of Geriatric Syndromes. NHANES: National Health and Nutrition Examination Survey. NHANES III: Third National Health and Nutrition Examination Survey. SABE: Salud, Bienestar y Envejecimiento. SEBAS: Social Environment and Biomarkers of Ageing Study. SGS: Sasaguri Genkimon Study. SLAS-II: Singapore Longitudinal Study of Aging-II. SNSAC: Swedish National Study on Aging and Care. SPPB: Short Physical Performance Battery. Sydney MAS: Sydney Memory and Ageing Study. THLHP: Tsimane Health & Life History Project. ‡YOITA: Yoita Longitudinal Study.

**Mobility test:** HGS: Habitual Gait Speed. TUG: Timed-Up and Go. UGT: Up and Go test. MWT: Metre walk test. minWT: Minute walk test. **Study design:** (no symbol) Cross-sectional analysis. * Longitudinal analysis. † Both Cross-sectional and Longitudinal analyses. **Country:** UK: United Kingdom. USA: United States of America. **NA:** Not applicable. **Analysis type:** BAS: Bivariate Analysis. MAS: Multivariate Analysis (only the sociodemographic predictors were reported).

**Supplementary File 4** Summary of study design and results (none of the included studies addressed religion)

| **First author surname, Year** | **Title** | **Age** | | **Gender** | | **Marital status** | | **Race** | | **Income** | | **Education** | | **Occupation** | | **Residence** | | **House ownership** | | **Social status** | |
| --- | --- | --- | --- | --- | --- | --- | --- | --- | --- | --- | --- | --- | --- | --- | --- | --- | --- | --- | --- | --- | --- |
|  |  | CSA | LA | CSA | LA | CSA | LA | CSA | LA | CSA | LA | CSA | LA | CSA | LA | CSA | LA | CSA | LA | CSA | LA |
| Al Snih, 2008 | Ethnic differences in physical performance in older Americans: data from the Third National Health and Nutrition Examination Survey (1988-1994) | MAS |  | MAS |  | N |  | TDS; MAS |  |  |  | MAS |  |  |  |  |  |  |  |  |  |
| Aoyagi, 2001 | Comparison of performance-based measures among native Japanese, Japanese-Americans in Hawaii and Caucasian women in the United States, ages 65 years and over: A cross-sectional study |  |  |  |  |  |  | TDS |  |  |  |  |  |  |  |  |  |  |  |  |  |
| Asher, 2012 | Most older pedestrians are unable to cross the road in time: a cross-sectional study | BAS; MAS |  | BAS; MAS |  |  |  |  |  |  |  | BAS |  |  |  |  |  |  |  |  |  |
| Barrera, 2017 | Associations between socioeconomic status, aging and functionality among older women |  |  |  |  |  |  |  |  |  |  | TDS |  |  |  |  |  |  |  |  |  |
| Bendall, 1989 | Factors affecting walking speed of elderly people | BAS |  | TDS |  |  |  |  |  |  |  |  |  |  |  |  |  |  |  |  |  |
| Binotto, 2019 | Gait speed associated factors in elderly subjects undergoing exams to obtain the driver's license Portuguese, English, Spanish | MAS |  | MAS |  |  |  |  |  |  |  |  |  |  |  |  |  |  |  |  |  |
| Blanco, 2012 | Racial differences in gait velocity in an urban elderly cohort |  |  | N |  |  |  | TDS: BAS: MAS |  |  |  |  |  |  |  |  |  |  |  |  |  |
| Bohannon, 1996 | Walking speed: Reference values and correlates for older adults | N |  | BAS;MAS |  |  |  |  |  |  |  |  |  |  |  |  |  |  |  |  |  |
| Bohannon, 2008 | Population representative gait speed and its determinants | BAS; MAS |  | BAS |  |  |  |  |  |  |  |  |  |  |  |  |  |  |  |  |  |
| Boulifard, 2019 | Home-based gait speed assessment: Normative data and racial/ethnic correlates among older adults | TDS; MAS |  | TDS; MAS |  |  |  | TDS; MAS |  | MAS |  | MAS |  |  |  | MAS |  |  |  |  |  |
| Brunner, 2009 | Social inequality in walking speed in early old age in the Whitehall II study | TDS |  | TDS |  | TDS |  | TDS |  | TDS |  |  |  |  |  |  |  |  |  | N |  |
| Buchner, 1996 | Evidence for a non-linear relationship between leg strength and gait speed | BAS |  | BAS |  |  |  |  |  |  |  |  |  |  |  |  |  |  |  |  |  |
| Busch, 2015 | Factors associated with lower gait speed among the elderly living in a developing country: a cross-sectional population-based study. | TDS; MAS |  | N |  |  |  | N |  |  |  | TDS; MAS |  |  |  |  |  |  |  |  |  |
| Butler, 2009 | Age and gender differences in seven tests of functional mobility | BAS |  | TDS |  |  |  |  |  |  |  |  |  |  |  |  |  |  |  |  |  |
| Carvalho de Abreu, 2021 | Functional performance of older adults: A comparison between men and women |  |  | N |  |  |  |  |  |  |  |  |  |  |  |  |  |  |  |  |  |
| ChilesShaffer, 2020 | The roles of body composition and specific strength in the relationship between race and physical performance in older adults | MAS |  |  |  |  |  | MAS |  | MAS |  | N |  |  |  |  |  |  |  |  |  |
| Coelho-Junior, 2021 | Age- and gender-related changes in physical function in community-dwelling Brazilian adults aged 50 to 102 years | TDS; BAS |  |  |  |  |  |  |  |  |  |  |  |  |  |  |  |  |  |  |  |
| Dommershuijsen, 2022 | Gait speed reference values in community-dwelling older adults - cross-sectional analysis from the Rotterdam Study | TDS |  | N |  |  |  |  |  |  |  | TDS |  |  |  |  |  |  |  |  |  |
| Fang, 2020 | Three-dimensional thoracic and pelvic kinematics and arm swing maximum velocity in older adults using inertial sensor system | TDS |  | N |  |  |  |  |  |  |  |  |  |  |  |  |  |  |  |  |  |
| Fiser, 2010 | Energetics of walking in elderly people: factors related to gait speed | N |  | TDS |  |  |  |  |  |  |  |  |  |  |  |  |  |  |  |  |  |
| Gomes, 2023 | Changes in physical performance among community-dwelling older adults in six years |  | C |  | C |  | C |  | N |  |  |  | C |  |  |  |  |  |  |  | TDS |
| Granic, 2018 | Factors associated with physical performance measures in a multiethnic cohort of older adults | MAS |  | MAS |  |  |  | TDS |  |  |  | MAS |  |  |  |  |  |  |  |  |  |
| Ibrahim, 2017 | ‘Timed Up and Go’ test: age, gender and cognitive impairment stratified normative values of older adults. | TDS; MAS |  | TDS; MAS |  |  |  |  |  |  |  |  |  |  |  |  |  |  |  |  |  |
| Idland, 2013 | Predictors of mobility in community-dwelling women aged 85 and older. |  | BAS; MAS |  |  |  |  |  |  |  |  |  | N |  |  |  |  |  |  |  |  |
| Iwakura, 2022 | Lower-limb muscle strength and major performance tests in community-dwelling older adults | MAS |  | N |  |  |  |  |  |  |  |  |  |  |  |  |  |  |  |  |  |
| Jerome, 2015 | Gait characteristics associated with walking speed decline in older adults: results from the Baltimore Longitudinal Study of Aging |  | MAS |  |  |  |  |  |  |  |  |  |  |  |  |  |  |  |  |  |  |
| Kamiya, 2019 | The 6-Minute Walk Test: Difference in explanatory variables for performance by community-dwelling older adults and patients hospitalized for cardiac disease | BAS; MAS |  | N |  |  |  |  |  |  |  |  |  |  |  |  |  |  |  |  |  |
| Lin, 2021 | Using hand grip strength to detect slow walking speed in older adults: the Yilan study | TDS; BAS; MAS |  | TDS; MAS |  |  |  |  |  |  |  | TDS; MAS |  |  |  |  |  |  |  |  |  |
| Lunar, 2019 | Mobility performance among community-dwelling older Filipinos who lived in urban and rural settings: A preliminary study |  |  |  |  |  |  |  |  |  |  |  |  |  |  | TDS |  |  |  |  |  |
| Makizako, 2017 | Age-dependent changes in physical performance and body composition in community-dwelling Japanese older adults | BAS; MAS |  | N |  |  |  |  |  |  |  |  |  |  |  |  |  |  |  |  |  |
| Mantel, 2019 | An investigation of the predictors of comfortable and fast gait speed in community-dwelling older adults | BAS |  | N |  |  |  |  |  |  |  |  |  |  |  |  |  |  |  |  |  |
| Milanović, 2013 | Age-related decrease in physical activity and functional fitness among elderly men and women. | TDS |  |  |  |  |  |  |  |  |  |  |  |  |  |  |  |  |  |  |  |
| Mohammed, 2021 | Influence of age, gender, and body mass index on balance and mobility performance in Indian community-dwelling older people | BAS |  | N |  |  |  |  |  |  |  |  |  |  |  |  |  |  |  |  |  |
| Payne, 2017 | Physical function in an aging population in rural South Africa: Findings from HAALSI and cross-national comparisons with HRS sister studies | TDS; MAS |  | TDS; MAS |  | MAS |  |  |  |  |  | N |  |  |  |  |  |  |  |  |  |
| Plouvier, 2016 | Socioeconomic disparities in gait speed and associated characteristics in early old age |  |  |  |  |  |  |  |  |  |  |  |  | MAS |  |  |  |  |  |  |  |
| Ramírez-Vélez, 2020 | Normative values for the short physical performance battery (SPPB) and their association with anthropometric variables in older Colombian adults. The SABE Study, 2015 | TDS; BAS; MAS |  | TDS; MAS |  |  |  |  |  |  |  |  |  |  |  |  |  |  |  |  |  |
| Rikli, 1999 | Functional fitness normative scores for community-residing older adults, ages 60–94. | TDS |  | TDS |  |  |  |  |  |  |  |  |  |  |  |  |  |  |  |  |  |
| Ruggero, 2013 | Gait speed correlates in a multiracial population of community-dwelling older adults living in Brazil: a cross-sectional population-based study | BAS; MAS |  | N |  |  |  |  |  | N |  | BAS |  |  |  |  |  |  |  |  |  |
| Schrack, 2012 | The role of energetic cost in the age-related slowing of gait speed | MAS |  | MAS |  |  |  |  |  |  |  |  |  |  |  |  |  |  |  |  |  |
| Seino, 2014 | Reference values and age and sex differences in physical performance measures for community-dwelling older Japanese: a pooled analysis of six cohort studies | TDS; MAS |  | TDS; MAS |  |  |  |  |  |  |  |  |  |  |  |  |  |  |  |  |  |
| Shubert, 2006 | Are scores on balance screening tests associated with mobility in older adults? | MAS |  | N |  |  |  |  |  |  |  |  |  |  |  |  |  |  |  |  |  |
| Sialino, 2019 | Sex differences in physical performance by age, educational level, ethnic groups and birth cohort: The Longitudinal Aging Study Amsterdam | MAS |  | TDS; MAS |  |  |  |  |  |  |  |  |  |  |  |  |  |  |  |  |  |
| Sialino, 2021 | The sex difference in gait speed among older adults: How do sociodemographic, lifestyle, social and health factors contribute? |  |  |  | TDS; MAS |  |  |  |  |  |  |  | N |  |  |  |  |  |  |  | MAS |
| Smolar, 2012 | Gait speed in community-dwelling African-American and Afro-Caribbean older adults | MAS |  | N |  |  |  | TDS; BAS; MAS |  |  |  | N |  |  |  |  |  |  |  |  |  |
| Sprague, 2023 [Tanzania] | Correlates of gait speed among older adults from 6 countries: Findings from the COSMIC collaboration | BAS |  | N |  |  |  |  |  |  |  | TDS |  |  |  |  |  |  |  |  |  |
| Sprague, 2023 [Nigeria] |  | BAS |  | TDS |  |  |  |  |  |  |  | N |  |  |  |  |  |  |  |  |  |
| Sprague, 2023 [South Korea] |  | BAS |  | N |  |  |  |  |  |  |  | TDS |  |  |  |  |  |  |  |  |  |
| Sprague, 2023 [Japan] |  | BAS |  | TDS |  |  |  |  |  |  |  | TDS |  |  |  |  |  |  |  |  |  |
| Sprague, 2023 [Singapore] |  | BAS |  | TDS |  |  |  |  |  |  |  | TDS |  |  |  |  |  |  |  |  |  |
| Sprague, 2023 [Australia] |  | BAS |  | TDS |  |  |  |  |  |  |  | TDS |  |  |  |  |  |  |  |  |  |
| Staples, 2020 | Examination of the correlation between physical and psychological measures in community-dwelling older adults | BAS; MAS |  |  |  |  |  |  |  |  |  | MAS |  |  |  |  |  |  |  |  |  |
| Tanaka, 2022 | Effects of age and gender on spatiotemporal and kinematic gait parameters in older adults | TDS |  | N |  |  |  |  |  |  |  |  |  |  |  |  |  |  |  |  |  |
| Tangen, 2020 | Measuring physical performance in highly active older adults: associations with age and gender? | BAS |  | N |  |  |  |  |  |  |  |  |  |  |  |  |  |  |  |  |  |
| Thaweewannakij, 2013 | Reference values of physical performance in Thai elderly people who are functioning well and dwelling in the community | TDS; MAS |  | TDS; MAS |  |  |  |  |  |  |  |  |  |  |  |  |  |  |  |  |  |
| Thompson, 1995 | Performance of community dwelling elderly on the timed up and go test | N |  | TDS |  |  |  |  |  |  |  |  |  |  |  |  |  |  |  |  |  |
| Thorpe, 2011 | Race, socioeconomic resources, and late-life mobility and decline: findings from the Health, Aging, and Body Composition study |  | MAS |  |  |  |  |  | TDS; MAS |  | MAS |  | MAS |  |  |  |  |  | MAS |  |  |
| Vasunilashorn, 2009 | Use of the Short Physical Performance Battery Score to predict loss of ability to walk 400 meters: analysis from the InCHIANTI study |  | MAS |  | N |  |  |  |  |  |  |  | N |  |  |  |  |  |  |  |  |
| Watson, 2010 | Executive function, memory, and gait speed decline in well-functioning older adults | TDS |  | TDS |  |  |  | TDS |  |  |  | TDS |  |  |  |  |  |  |  |  |  |
| Welmer, 2013 | Education-related differences in physical performance after age 60: A cross-sectional study assessing variation by age, gender and occupation. | TDS |  | TDS |  |  |  |  |  |  |  | TDS; MAS |  | TDS |  |  |  |  |  |  |  |
| Wheaton, 2016 [USA] | Female disability disadvantage: A global perspective on sex differences in physical function and disability. |  |  | MAS |  |  |  |  |  |  |  |  |  |  |  |  |  |  |  |  |  |
| Wheaton, 2016 [Taiwan] |  |  |  | MAS |  |  |  |  |  |  |  |  |  |  |  |  |  |  |  |  |  |
| Wheaton, 2016 [China] |  |  |  | MAS |  |  |  |  |  |  |  |  |  |  |  |  |  |  |  |  |  |
| Wheaton, 2016 [Bolivia] |  |  |  | MAS |  |  |  |  |  |  |  |  |  |  |  |  |  |  |  |  |  |
| Wu, 2021 | Associations between functional fitness and walking speed in older adults | MAS |  |  |  |  |  |  |  |  |  |  |  |  |  |  |  |  |  |  |  |
| Yaoxin, 2022 | Mediating effect of lower extremity muscle strength on the relationship between mobility and cognitive function in Chinese older adults: A cross-sectional study | TDS; BAS |  | N |  |  |  |  |  |  |  | TDS; BAS |  | N |  |  |  |  |  |  |  |

Younger age, men, married, urban area, higher income, higher education, non-manual job, religious, higher social status, homeowner, or Caucasian = test of differences = statistically significant higher mobility = TD**S**

Younger age, men, married, urban area, higher income, higher education, non-manual job, religious, higher social status, homeowner, or Caucasian = bivariate association/correlation = statistically significant higher mobility = BAS

Younger age, men, married, urban area, higher income, higher education, non-manual job, religious, higher social status, homeowner, or Caucasian = multivariate association = statistically significant higher mobility = MAS

Older age, women, not married, rural area, lower income, lower education, manual job, irreligious, lower social status, non-homeowner, non-Caucasian = test of differences = statistically significant higher mobility = TD**S**

Older age, women, not married, rural area, lower income, lower education, manual job, irreligious, lower social status, non-homeowner, non-Caucasian = bivariate association/correlation = statistically significant higher mobility = BAS

Older age, women, not married, rural area, lower income, lower education, manual job, irreligious, lower social status, non-homeowner, non-Caucasian = multivariate association = statistically significant higher mobility = MAS

Result not statistically significant = **N**

Result of different mobility tests conflicting = **C**

CSA = cross-sectional analysis. LA = longitudinal analysis.

**Supplementary File 4** Risk of Bias Assessment for all studies (*n* = 57)

| **Author, Year** | **Title** | **Joanna Briggs Institute’s appraisal checklist for analytic cross-sectional studies** | | | | | | | | | |
| --- | --- | --- | --- | --- | --- | --- | --- | --- | --- | --- | --- |
|  |  | **Item 1** | **Item 2** | **Item 3** | **Item 4** | **Item 5** | **Item 6** | **Item 7** | **Item 8** | **Score** | **Risk** |
| Al Snih, 2008 | Ethnic differences in physical performance in older Americans: data from the Third National Health and Nutrition Examination Survey (1988-1994) | Yes | Yes | Yes | Yes | Yes | Yes | Yes | Yes | 8 | Low |
| Aoyagi, 2001 | Comparison of performance-based measures among native Japanese, Japanese-Americans in Hawaii and Caucasian women in the United States, ages 65 years and over: A cross-sectional study | Yes | Yes | Yes | Yes | Yes | Yes | Yes | Yes | 8 | Low |
| Asher, 2012 | Most older pedestrians are unable to cross the road in time: a cross-sectional study | Unclear | Unclear | Unclear | Yes | Yes | No | Yes | Yes | 4 | Medium |
| Barrera, 2017 | Associations between socioeconomic status, aging and functionality among older women | Unclear | Yes | Yes | Yes | Yes | No | Yes | Yes | 6 | Low |
| Bendall, 1989 | Factors affecting walking speed of elderly people | Unclear | No | Yes | Yes | Yes | Yes | Yes | Yes | 6 | Low |
| Binotto, 2019 | Gait speed associated factors in elderly subjects undergoing exams to obtain the driver's license Portuguese, English, Spanish | Yes | No | Yes | Yes | No | No | Yes | No | 4 | Medium |
| Blanco, 2012 | Racial differences in gait velocity in an urban elderly cohort | Yes | Yes | Yes | Yes | Yes | Yes | Yes | Yes | 8 | Low |
| Bohannon, 1996 | Walking speed: Reference values and correlates for older adults | Yes | No | Yes | Yes | Unclear | Yes | Yes | Yes | 6 | Low |
| Bohannon, 2008 | Population representative gait speed and its determinants | Yes | Yes | Yes | Yes | Yes | Yes | Yes | Yes | 8 | Low |
| Boulifard, 2019 | Home-based gait speed assessment: Normative data and racial/ethnic correlates among older adults | Yes | Yes | Yes | Yes | Yes | Yes | Yes | Yes | 8 | Low |
| Brunner, 2009 | Social inequality in walking speed in early old age in the Whitehall II study | No | Yes | Yes | Yes | Yes | Yes | Yes | Unclear | 6 | Low |
| Buchner, 1996 | Evidence for a non-linear relationship between leg strength and gait speed | Yes | Yes | Yes | Yes | Yes | Yes | Yes | Yes | 8 | Low |
| Busch,, 2015 | Factors associated with lower gait speed among the elderly living in a developing country: a cross-sectional population-based study. | Yes | Yes | Yes | Yes | Yes | Yes | Yes | Unclear | 7 | Low |
| Butler, 2009 | Age and gender differences in seven tests of functional mobility | Unclear | Yes | Yes | Yes | Unclear | No | Yes | Yes | 5 | Medium |
| Carvalho de Abreu, 2021 | Functional performance of older adults: A comparison between men and women | Yes | Yes | Yes | Yes | Yes | Yes | Yes | Unclear | 7 | Low |
| ChilesShaffer, 2020 | The roles of body composition and specific strength in the relationship between race and physical performance in older adults | Yes | Yes | Yes | Yes | Yes | Yes | Yes | Yes | 8 | Low |
| Coelho-Junior, 2021 | Age- and gender-related changes in physical function in community-dwelling Brazilian adults aged 50 to 102 years | Yes | Yes | Yes | Yes | Unclear | Unclear | Yes | Yes | 6 | Low |
| Dommershuijsen, 2022 | Gait speed reference values in community-dwelling older adults - cross-sectional analysis from the Rotterdam Study | Yes | Yes | Yes | Yes | No | No | Yes | Yes | 6 | Low |
| Fang, 2020 | Three-dimensional thoracic and pelvic kinematics and arm swing maximum velocity in older adults using inertial sensor system | Yes | Yes | Yes | Yes | No | No | Yes | Yes | 6 | Low |
| Fiser, 2010 | Energetics of walking in elderly people: factors related to gait speed | Yes | Yes | Unclear | Yes | No | No | Unclear | Yes | 4 | Medium |
| Gomes, 2023 | Changes in physical performance among community-dwelling older adults in six years | Yes | Yes | Yes | Yes | No | No | Yes | Yes | 6 | Low |
| Granic, 2018 | Factors associated with physical performance measures in a multiethnic cohort of older adults | Yes | Yes | No | Yes | Yes | Yes | Yes | Yes | 7 | Low |
| Ibrahim, 2017 | ‘Timed Up and Go’ test: age, gender and cognitive impairment stratified normative values of older adults. | Yes | Yes | Yes | Yes | Yes | Yes | Yes | Yes | 8 | Low |
| Idland, 2013 | Predictors of mobility in community-dwelling women aged 85 and older. | Yes | Yes | Yes | Yes | Yes | Yes | Yes | Yes | 8 | Low |
| Iwakura, 2022 | Lower-limb muscle strength and major performance tests in community-dwelling older adults | Yes | Yes | Yes | Yes | Yes | Unclear | Yes | Yes | 7 | Low |
| Jerome, 2015 | Gait characteristics associated with walking speed decline in older adults: results from the Baltimore Longitudinal Study of Aging | Yes | Yes | Yes | Yes | Yes | Unclear | Yes | Yes | 7 | Low |
| Kamiya, 2019 | The 6-Minute Walk Test: Difference in explanatory variables for performance by community-dwelling older adults and patients hospitalized for cardiac disease | Unclear | Yes | Yes | Yes | Unclear | Unclear | Yes | Yes | 5 | Medium |
| Lin, 2021 | Using hand grip strength to detect slow walking speed in older adults: the Yilan study | Yes | Yes | Yes | Yes | Yes | Yes | Yes | Yes | 8 | Low |
| Lunar, 2019 | Mobility performance among community-dwelling older Filipinos who lived in urban and rural settings: A preliminary study | Yes | Yes | Yes | Yes | Yes | Yes | Yes | Yes | 8 | Low |
| Makizako, 2017 | Age-dependent changes in physical performance and body composition in community-dwelling Japanese older adults | Yes | Yes | Yes | Yes | No | No | Yes | Yes | 6 | Low |
| Mantel, 2019 | An investigation of the predictors of comfortable and fast gait speed in community-dwelling older adults | Yes | Yes | Yes | Yes | Yes | Yes | Yes | Yes | 8 | Low |
| Milanović, 2013 | Age-related decrease in physical activity and functional fitness among elderly men and women. | Yes | Yes | Yes | Yes | Yes | Yes | Yes | Yes | 8 | Low |
| Mohammed, 2021 | Influence of age, gender, and body mass index on balance and mobility performance in Indian community-dwelling older people | Yes | Yes | Yes | Yes | No | No | Yes | Yes | 6 | Low |
| Payne, 2017 | Physical function in an aging population in rural South Africa: Findings from HAALSI and cross-national comparisons with HRS sister studies | Yes | Yes | Yes | Yes | Yes | Yes | Yes | Yes | 8 | Low |
| Plouvier, 2016 | Socioeconomic disparities in gait speed and associated characteristics in early old age | Yes | Yes | Yes | Yes | Yes | Yes | Yes | Yes | 8 | Low |
| Ramírez-Vélez, 2020 | Normative values for the short physical performance battery (SPPB) and their association with anthropometric variables in older Colombian adults. The SABE Study, 2015 | No | Yes | Yes | Yes | Yes | Unclear | Yes | Yes | 6 | Low |
| Rikli , 1999 | Functional fitness normative scores for community-residing older adults, ages 60–94. | Yes | Yes | Yes | Yes | Yes | Yes | Yes | Yes | 8 | Low |
| Ruggero, 2013 | Gait speed correlates in a multiracial population of community-dwelling older adults living in Brazil: a cross-sectional population-based study | Yes | Yes | Yes | Yes | Unclear | Unclear | Yes | Yes | 6 | Low |
| Schrack, 2012 | The role of energetic cost in the age-related slowing of gait speed | Yes | Yes | Yes | Yes | Yes | Yes | Yes | Yes | 8 | Low |
| Seino, 2014 | Reference values and age and sex differences in physical performance measures for community-dwelling older Japanese: a pooled analysis of six cohort studies | Yes | Yes | Yes | Yes | Yes | Yes | Yes | Yes | 8 | Low |
| Shubert, 2006 | Are scores on balance screening tests associated with mobility in older adults? | Yes | Yes | Yes | Yes | Yes | Yes | Yes | Yes | 8 | Low |
| Sialino, 2019 | Sex differences in physical performance by age, educational level, ethnic groups and birth cohort: The Longitudinal Aging Study Amsterdam | Unclear | Yes | Yes | Yes | Yes | Yes | Yes | Yes | 7 | Low |
| Sialino, 2021 | The sex difference in gait speed among older adults: How do sociodemographic, lifestyle, social and health factors contribute? | Unclear | Yes | Yes | Yes | Yes | Yes | Yes | Yes | 6 | Low |
| Smolar, 2012 | Gait speed in community-dwelling African-American and Afro-Caribbean older adults | Yes | Yes | Yes | Yes | Yes | Yes | Yes | Yes | 8 | Low |
| Sprague, 2023 | Correlates of gait speed among older adults from 6 countries: Findings from the COSMIC collaboration | Yes | Yes | Yes | Yes | No | No | Yes | Yes | 6 | Low |
| Staples, 2020 | Examination of the correlation between physical and psychological measures in community-dwelling older adults | Yes | Yes | Yes | Yes | Unclear | Unclear | Yes | Unclear | 5 | Medium |
| Tanaka, 2022 | Effects of age and gender on spatiotemporal and kinematic gait parameters in older adults | Yes | Yes | Yes | Yes | No | No | Yes | Yes | 6 | Low |
| Tangen, 2020 | Measuring physical performance in highly active older adults: associations with age and gender? | Yes | Yes | Yes | Yes | Yes | Yes | Yes | Yes | 8 | Low |
| Thaweewannakij, 2013 | Reference values of physical performance in Thai elderly people who are functioning well and dwelling in the community | Yes | Yes | Yes | Yes | Unclear | Unclear | Yes | Yes | 6 | Low |
| Thompson, 1995 | Performance of community dwelling elderly on the timed up and go test | Yes | Yes | Yes | Yes | Yes | Yes | Yes | Yes | 8 | Low |
| Thorpe, 2011 | Race, socioeconomic resources, and late-life mobility and decline: findings from the Health, Aging, and Body Composition study | Yes | Yes | Yes | Yes | Yes | Yes | Yes | Yes | 8 | Low |
| Vasunilashorn, 2009 | Use of the Short Physical Performance Battery Score to predict loss of ability to walk 400 meters: analysis from the InCHIANTI study | Unclear | Yes | Yes | Yes | Yes | Unclear | Yes | Yes | 6 | Low |
| Watson, 2010 | Executive function, memory, and gait speed decline in well-functioning older adults | Yes | Yes | Yes | Yes | Yes | Yes | Yes | Yes | 8 | Low |
| Welmer, 2013 | Education-related differences in physical performance after age 60: A cross-sectional study assessing variation by age, gender and occupation. | Yes | Yes | Yes | Yes | Yes | Yes | Yes | Yes | 8 | Low |
| Wheaton, 2016 | Female disability disadvantage: A global perspective on sex differences in physical function and disability. | Yes | Yes | Yes | Yes | Yes | Yes | Yes | Yes | 8 | Low |
| Wu, 2021 | Associations between functional fitness and walking speed in older adults | Yes | Yes | Yes | Yes | Yes | Yes | Yes | Yes | 8 | Low |
| Yaoxin, 2022 | Mediating effect of lower extremity muscle strength on the relationship between mobility and cognitive function in Chinese older adults: A cross-sectional study | Yes | Yes | Unclear | Yes | Yes | Yes | Yes | Yes | 7 | Low |

Item 1: Were the criteria for inclusion in the sample clearly defined? Item 2: Were the study subjects and the setting described in detail? Item 3: Was the exposure measured in a valid and reliable way? Item 4: Were objective, standard criteria used for measurement of the condition? Item 5: Were confounding factors identified? Item 6: Were strategies to deal with confounding factors stated? Item 7: Were the outcomes measured in a valid and reliable way? Item 8: Was appropriate statistical analysis used?
